# A universal framework for the quantum simulation of Yang–Mills theory

**DOI:** 10.1038/s42005-025-02421-6

**Published:** 2026-02-19

**Authors:** Jad C. Halimeh, Masanori Hanada, Shunji Matsuura, Franco Nori, Enrico Rinaldi, Andreas Schäfer

**Affiliations:** 1 Max Planck Institute of Quantum Optics, Garching, Germany; 2 Department of Physics and Arnold Sommerfeld Center for Theoretical Physics (ASC) Ludwig Maximilian, University of Munich, Munich, Germany; 3 Munich Center for Quantum Science and Technology (MCQST), Munich, Germany; 4 School of Mathematical Sciences, Queen Mary University of London Mile End Road, London, United Kingdom; 5 qBraid Co., Chicago, IL USA; 6 Interdisciplinary Theoretical & Mathematical Science Program (iTHEMS) RIKEN, Wako, Saitama Japan; 7 Department of Electrical and Computer Engineering, University of British Columbia, Vancouver, BC Canada; 8 Department of Physics, University of Guelph, Guelph, ON Canada; 9 Center for Mathematical Science and Advanced Technology, Japan Agency for Marine-Earth Science and Technology, Yokohama, Japan; 10 Center for Quantum Computing (RQC), RIKEN, Wako, Saitama Japan; 11 Physics Department, University of Michigan, Ann Arbor, MI USA; 12 Quantinuum K.K., Chiyoda-ku, Tokyo Japan; 13 Institute of Theoretical Physics, University of Regensburg, Regensburg, Germany

**Keywords:** Physics, Theoretical particle physics

## Abstract

Quantum computers promise to simulate complex quantum field theories that are intractable for classical computers, potentially revealing new physics in strongly interacting systems. Current approaches for simulating Yang-Mills gauge theories face significant technical barriers due to complex group structures and complicated truncation schemes that vary drastically between different theories. Here we show that the orbifold lattice formulation provides a universal framework for quantum simulation of Yang-Mills theories with arbitrary gauge groups and dimensions. Our approach reduces all theories to the same simple Hamiltonian form, enabling implementation with standard quantum gates regardless of system complexity. We demonstrate explicit quantum circuits using only controlled-NOT and single-qubit operations, with concrete resource estimates for time evolution algorithms. This universal framework simplifies quantum simulation of gauge theories and enables systematic scaling to larger systems on fault-tolerant quantum computers.

## Abstract (web summary)

The quantum simulation of lattice gauge theories is anticipated to be an important scientific application of future quantum computing capabilities. This work elaborates on a formulation of lattice gauge theory quantum simulation that aims to require quantum computing techniques akin to those for simulating *ϕ*^4^ scalar field theory by utilizing non-compact continuous variable quantum degrees of freedom.

## Introduction

Recent advances in quantum error correction have made the prospect of fault-tolerant quantum computing ever more promising. A very exciting prospect of achieving the latter lies in quantum-simulating the holy grail of high-energy physics, QCD^1–9^. For example, this would provide a complementary venue to dedicated particle colliders for the investigation of QCD, aiding in unraveling many of its outstanding mysteries^10,11^.

One of the first steps to realize this potential is to write the QCD Hamiltonian explicitly in a form that can be implemented on digital universal quantum computers. The standard method is to replace the infinite-volume continuum space with a finite-size lattice, in such a way that the continuum and large-volume result can be obtained systematically by sending the lattice size to infinity^12^. Furthermore, because gluons are bosons, we need to truncate the Hilbert space of the lattice theory by introducing a certain truncation level *Λ* and taking the limit *Λ* → *∞*. We must write the truncated Hamiltonian explicitly for arbitrary lattice size and arbitrary truncation level, in such a way that the implementation on quantum computers is straightforward.

QCD is a Yang–Mills theory with an SU(3) gauge group coupled to fermions in the fundamental representation^13^. The QCD effects are typically the least well-understood for Standard Model processes and thus limit the theoretical precision reached, especially for time-dependent genuinely non-perturbative processes that cannot be treated by either perturbative QCD (pQCD) or Lattice QCD (LQCD). Currently, phenomenological models using various approximations are used to glean, e.g., information about intermediate-time far-from-equilibrium highly non-perturbative quantum processes underlying the formation of the Quark-gluon plasma. Quantum simulation offers the unique prospect of probing such processes from a first-principles standpoint, providing snapshots of this dynamics that can yield deep insights into outstanding questions^14^. Furthermore, a major driving force in particle physics is to find and investigate physics beyond the Standard Model, e.g., quantum gravity, for which a plethora of suggestions have been made, typically involving different gauge groups, additional symmetries, or novel interaction terms^15^.

Thus, to fully profit from the possibilities opened up by future, fault-tolerant quantum computing, it will be crucial to develop universal formulations that can easily be adapted to any member of large groups of theories. (In principle, any formulation with the correct continuum limit is eligible.) For example, the large-*N* limit of SU(*N*) gauge theories plays a prominent role because it allows us to obtain exact analytic results^16,17^. Other examples are, e.g., SU(5) and SO(10) candidates for Grand Unified gauge theories. Therefore, the study of SU(*N*) Yang–Mills theory with *N* ≥ 3 is a promising starting point, covering QCD as well as many models beyond the Standard Model.

As an almost trivial but important remark, we note that Yang–Mills theory and QCD are merely a small fraction of many important problems. It is presently hoped that quantum computing will allow us to solve a long list of computational problems for which classical computers are inefficient, and this list is expected to only get longer with time^18^. To meet all these expectations, one will need versatile codes that allow treating many of these problems without the need to undertake quantum code development for each of them from scratch. The situation will thus be quite different from what it is now, where development focuses on a few, highly specific applications, and invests most work on highly specific resource optimization using, e.g., special properties of the chosen problem that do not generalize to other problems of actual interest. It may be better *not* to rely on special properties such as the simplicity of the representation theory for U(1) or SU(2), or perhaps, any features specific to Yang–Mills theory, so that we can utilize the power of more generic methods developed by the wide research community.

Currently, the most popular choice of lattice Hamiltonian for SU(*N*) Yang–Mills theory within the high-energy physics community is the Kogut–Susskind formulation^19^. This is the Hamiltonian version of Wilson’s Lagrangian formulation^20^ that uses unitary link variables. Specifically, there are unitary link operators ${\hat{U}}_{j,\overrightarrow{x}}$ living on a link connecting lattice site $\overrightarrow{x}$ and $\overrightarrow{x}+\hat{j}$, where *j* = 1, 2, 3 are spatial dimensions and $\hat{j}$ is the unit vector along the *j*th direction. In addition, the conjugate momenta ${\hat{E}}_{j,\overrightarrow{x}}$ are introduced. To describe the Hilbert space, one can use the coordinate basis (also called magnetic basis) or the momentum basis (also called electric basis). The coordinate basis uses the coordinate eigenstates $\left\vert U\right\rangle$ that satisfies ${\hat{U}}_{j,\overrightarrow{x}}\left\vert U\right\rangle ={U}_{j,\overrightarrow{x}}\left\vert U\right\rangle$, where ${U}_{j,\overrightarrow{x}}$ is an *N* × *N* special unitary matrix. For a quantum state $\left\vert \Phi \right\rangle$, the wavefunction $\Phi (U)=\left\langle U\right\vert \left\vert \Phi \right\rangle$ is defined on ${\prod }_{j,\overrightarrow{x}}{[{{\rm{SU}}}(N)]}_{j,\overrightarrow{x}}$, where ${[{{\rm{SU}}}(N)]}_{j,\overrightarrow{x}}$ is the SU(*N*) group manifold corresponding to the link between $\overrightarrow{x}$ and $\overrightarrow{x}+\hat{j}$. It is a nontrivial task to truncate the SU(*N*) group manifold systematically so that the truncation effect can be evaluated straightforwardly and at the same time ${\hat{E}}_{j,\overrightarrow{x}}$ takes a simple form. The momentum basis uses the SU(*N*)-analog of the Fourier transform defined by the Peter–Weyl theorem^21,22^. This requires complicated group theory, specifically the knowledge of all irreducible representations and their Clebsch–Gordan coefficients. Although the momentum basis allows, in principle, a systematic truncation, as shown in a pioneering paper by Byrnes and Yamamoto^23^, it is technically complicated except for special cases such as SU(2). Whether we use the coordinate basis or the momentum basis, it is nontrivial to write down the truncated Hamiltonian, particularly for *N* ≥ 3. To get some intuition for the level of complications, see the relatively simple cases of the coordinate basis for SU(2)^24,25^, Fourier transform for a few discrete subgroups of SU(3)^26^, the use of q-deformation^27–29^, and some simplifications in the large-*N* limit^30^.

It is fair to say that it is very challenging to program SU(*N*) Yang–Mills theory with *N* ≥ 3 in 2 + 1 or 3 + 1 on a quantum computer using the Kogut–Susskind Hamiltonian; starting already with the simplest task of writing down the explicit Hamiltonian in terms of Pauli strings. Perhaps it is possible to write down the Hamiltonian explicitly either on the momentum basis or coordinate basis by using automated computer algebra systems, but still, there is no clear path to resolve other issues associated with the complicated expressions. Indeed, currently the only known large-scale experimental realizations of lattice gauge theories on quantum hardware are either in 1 + 1^31,32^ or 2 + 1 dimensions^33–35^, with a two-level representation of an Abelian gauge field. This motivates efforts to find an alternative lattice formulation that is straightforward to generalize to any dimension and gauge group. We suggest choosing the orbifold lattice formulation which does not suffer from these technical complications because of its use of non-compact complex link variables ${Z}_{j,\overrightarrow{x}}$ instead of compact unitary link variables ${U}_{j,\overrightarrow{x}}$^36,37^.

Quantum simulations of a matrix model and a gauge theory using the orbifold lattice formulation are very similar. Matrix models are interesting in their own right for many reasons, most notably as a non-perturbative definition of quantum gravity via gauge/gravity duality^38^. Therefore, by understanding how matrix models and orbifold lattice gauge theories can be studied on quantum computers, we can approach many important problems including QCD and quantum gravity.

In this work, we study SU(*N*) Yang–Mills theories on orbifold lattices and SU(*N*) Hermitian matrix models within a universal framework for quantum simulation. We show that both classes of theories can be formulated using standard bosonic variables with simple kinetic and quartic potential terms, so that their Hamiltonians can be written in a compact, universal form,

1 $$\hat{H}=\frac{1}{2}\sum\limits_{a}{\hat{p}}_{a}^{2}+V(\hat{x})\,,$$

where $V(\hat{x})$ is at most quartic. This enables direct mapping to Pauli strings, efficient implementation on digital quantum computers, and unified resource estimates for qubits and gate counts. Our results establish a broadly applicable approach that simplifies the study of gauge theories and matrix models on quantum hardware, laying the foundation for future exploration of QCD, beyond-the-Standard-Model physics, and quantum gravity.

## Results

### Basic idea

As we will see in section “Matrix models” and section “Orbifold lattice”, the Hamiltonians of matrix model and orbifold lattice gauge theory are schematically written as (1), where $V(\hat{x})$ is at most a fourth-order polynomial. Therefore, we discuss how a Hamiltonian for *N*_*b*_ bosons of the form

2 $$\hat{H}=\sum\limits_{a}\frac{1}{2}{\hat{p}}_{a}^{2}+\sum\limits_{a,b,c,d}{C}_{abcd}\,{\hat{x}}_{a}{\hat{x}}_{b}{\hat{x}}_{c}{\hat{x}}_{d}\,,$$

where *C*_*abcd*_ is an arbitrary real number, can be simulated on a quantum device. In this section, we focus on getting a simple truncated Hamiltonian, postponing an explicit construction of a quantum circuit for the Hamiltonian time evolution until the section “Resource estimate for Suzuki–Trotter time evolution”.

The potential part and kinetic part of the Hamiltonian become simple in the coordinate basis and momentum basis, respectively. Unlike in the Kogut–Susskind formulation, the Fourier transform between these two bases is straightforward for a generic gauge group SU(*N*). Truncation can be performed in a way compatible with the quantum Fourier transform.

By setting *N*_*b*_ = 1, the summations in (2) reduce to single terms, and the interaction part can be simplified until we arrive at the simplest nontrivial example, a single quantum anharmonic oscillator,

3 $$\hat{H}=\frac{{\hat{p}}^{2}}{2}+\frac{{\hat{x}}^{4}}{4}\,.$$

We will comment on this example in the section “Discussion”. We are optimistic that, by then, readers will see how this example captures the essence of our approach and serves as an excellent starting point for quantum simulations of more intriguing systems.

We begin by explaining truncation in the coordinate basis. Let us use $\{\left\vert \overrightarrow{x}\right\rangle \}$ to denote all *N*_*b*_ bosons simultaneously. By using this expression, we mean that the coordinate eigenstate of the system is

4 $$\left\vert \overrightarrow{x}\right\rangle ={\otimes }_{a}\left\vert {x}_{a}\right\rangle \,,$$

where each boson has coordinate eigenstate $\left\vert {x}_{a}\right\rangle$ (*a* = 1, 2, ⋯,  *N*_*b*_). The coordinate eigenstate $\left\vert \overrightarrow{x}\right\rangle$ is defined b

5 $$\hat{\overrightarrow{x}}\left\vert \overrightarrow{x}\right\rangle =\overrightarrow{x}\left\vert \overrightarrow{x}\right\rangle .$$

Moreover, we consider the system Hilbert space as a tensor product of the Hilbert spaces of the individual bosons in the coordinate eigenbasis. This Hilbert space ${{\mathcal{H}}}$ corresponds to the extended Hilbert space ${{{\mathcal{H}}}}_{{{\rm{ext}}}}$ introduced in later sections.

6 $${{\mathcal{H}}}={\otimes }_{a}{{{\mathcal{H}}}}_{a}, \quad {{{\mathcal{H}}}}_{a}={{\rm{Span}}}\{\left\vert {x}_{a}\right\rangle | {x}_{a}\in {\mathbb{R}}\}\,.$$

**Fig. 1:**
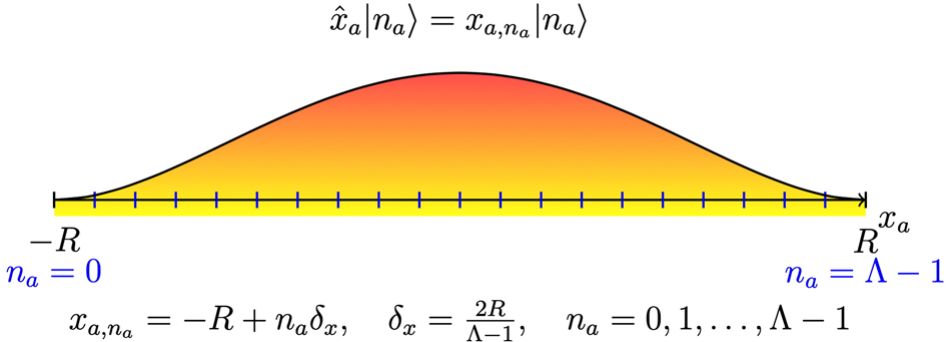
Discretization of a bosonic coordinate. The coordinate operator $\hat{x}a$ acts on a limited number of different states $\left\vert {n}_{a}\right\rangle$ with discretized eigenvalues ${x}_{a,{n}_{a}}$, labeled by integers *n*_*a*_. The gradient shading represents a possible wavefunction realization for this single bosonic degree of freedom.

So far, each boson has a wavefunction that can be represented in the basis given by $\left\vert {x}_{a}\right\rangle$, which lives in an infinite-dimensional Hilbert space. For example, one can imagine a one-dimensional quantum oscillator being in a superposition of many coordinates/positions. In order to reduce the problem to a finite-dimensional Hilbert space, for each boson coordinate *x*_*a*_, we introduce a cutoff,

7 $$-R\le {x}_{a}\le R\,,$$

and discretize *x*_*a*_ by introducing *Λ*≥2 points, with a slight modification of *x*_*a*,*n*_ and *δ*_*x*_ required when periodic boundary conditions are imposed, as we will see shortly and as depicted in Fig. 1.

8 $${x}_{a,{n}_{a}}=-R+{n}_{a}{\delta }_{x}\,,\qquad {\delta }_{x}=\frac{2R}{\Lambda -1}\,,\qquad {n}_{a}=0,1,\cdots \,,\Lambda -1\,.$$

We consider *Λ*, *δ*_*x*_, and *R* as truncation parameters that can and need to be adjusted. In particular, they each have limiting values that should be reached in order to recover the original infinite-dimensional Hilbert space: *Λ* should be sent to *∞*, together with *R*, while *δ*_*x*_ goes to 0.

By using $\left\vert {n}_{a}\right\rangle$ to denote $\left\vert {x}_{a,{n}_{a}}\right\rangle$, we can write the operator ${\hat{x}}_{a}$ acting diagonally on ${{{\mathcal{H}}}}_{a}$ as

9 $${\hat{x}}_{a}={\sum }_{{n}_{a}=0}^{\Lambda -1}{x}_{a,{n}_{a}}\left\vert {n}_{a}\right\rangle \left\langle {n}_{a}\right\vert =-R\cdot {{\bf{1}}}+{\delta }_{x}\cdot {\hat{n}}_{a}\,,$$

where

10 $${\hat{n}}_{a}\equiv \sum\limits_{{n}_{a}}{n}_{a}\left\vert {n}_{a}\right\rangle \left\langle {n}_{a}\right\vert $$

is the bosonic number operator. This operator can be extended to the operator acting on ${{\mathcal{H}}}$, assuming that it acts as the identity on ${{{\mathcal{H}}}}_{{a}^{{\prime} }}$ for ${a}^{{\prime} }\ne a$.

We then write ${\hat{n}}_{a}$ as a sum of Pauli operators acting on *Q* qubits representing a number of states equal to the number of points *Λ* = 2^*Q*^. Using the binary form with *b*_*a*,*i*_ = {0, 1},

11 $$\left\vert {n}_{a}\right\rangle =\left\vert {b}_{a,1}\right\rangle \left\vert {b}_{a,2}\right\rangle \cdots \left\vert {b}_{a,Q}\right\rangle \,,\qquad {n}_{a}={b}_{a,1}+2{b}_{a,2}\cdots +{2}^{Q-1}{b}_{a,Q}\,,$$

the number operator can be written by using Pauli *σ*_*z*_ gates,

12 $${\hat{n}}_{a} = 	 - \frac{{\hat{\sigma }}_{z;a,1}-{{\bf{1}}}}{2}-2\cdot \frac{{\hat{\sigma }}_{z;a,2}-{{\bf{1}}}}{2}-\cdots -{2}^{Q-1}\cdot \frac{{\hat{\sigma }}_{z;a,Q}-{{\bf{1}}}}{2} \\ = 	 - \frac{{\hat{\sigma }}_{z;a,1}}{2}-2\cdot \frac{{\hat{\sigma }}_{z;a,2}}{2}-\cdots -{2}^{Q-1}\cdot \frac{{\hat{\sigma }}_{z;a,Q}}{2}+\frac{\Lambda -1}{2}\cdot {{\bf{1}}},$$

where ${\hat{\sigma }}_{z;a,i}$ is the Pauli ${\hat{\sigma }}_{z}$ operator acting on $\left\vert {b}_{a,i}\right\rangle$. Note that our convention is

13 $${\sigma }_{z}\equiv \left\vert 0\right\rangle \left\langle 0\right\vert -\left\vert 1\right\rangle \left\langle 1\right\vert \,.$$

Therefore,

14 $${\hat{x}}_{a}=-{\delta }_{x}\cdot \left(\frac{{\hat{\sigma }}_{z;a,1}}{2}+2\cdot \frac{{\hat{\sigma }}_{z;a,2}}{2}+\cdots +{2}^{Q-1}\cdot \frac{{\hat{\sigma }}_{z;a,Q}}{2}\right)\,.$$

There are many four-boson couplings of the form ${\hat{x}}_{a}\otimes {\hat{x}}_{b}\otimes {\hat{x}}_{c}\otimes {\hat{x}}_{d}$. Each $\hat{x}$ is a sum of ${\hat{\sigma }}_{z,1}$,..., ${\hat{\sigma }}_{z,Q}$. Therefore, each four-boson coupling consists of *Q*^4^ terms, each of which is a tensor product of four ${\hat{\sigma }}_{z}$’s. If the same boson appears more than once in a given coupling, e.g., ${\hat{x}}_{a}^{2}{\hat{x}}_{b}^{2}$, then terms with fewer than four ${\hat{\sigma }}_{z}$’s also appear. The same structure is already present for the harmonic oscillator (3) and was explicitly studied in ref. ^39^, and much earlier in ref. ^40^.

Next, we discuss the implementation of the periodic boundary condition. Technically, it is convenient to use the periodic boundary condition *x* + 2*R* ~ *x*. In this case, a convenient convention is to take

15 $${\delta }_{x}=\frac{2R}{\Lambda }$$

and

16 $${x}_{a,{n}_{a}}=-\frac{\Lambda -1}{\Lambda }R+{n}_{a}{\delta }_{x}=\left({n}_{a}-\frac{\Lambda -1}{2}\right){\delta }_{x}\,.$$

Hence, ${x}_{a,{n}_{a}}$ takes values $\pm \frac{{\delta }_{x}}{2}$, $\pm \frac{3{\delta }_{x}}{2}$, ..., $\pm \frac{(\Lambda -1){\delta }_{x}}{2}$. To reduce the truncation effect, we must take *R* large enough for *x* ~ ± *R* not to be significantly excited, such that the boundary condition does not affect the physics of interest. In other words: if the boundary condition matters, *R* is not large enough. The study of truncation effects is usually specific for the system under study, including its parameters, such as the coupling constant. It is often the case that these systematic effects need to be studied numerically. An example targeting expectation values computed via classical sampling methods was reported in ref. ^39^.

Next, we introduce the quantum Fourier transform and the corresponding momentum basis. With the periodic boundary condition, the shift operator ${\hat{S}}_{a}\equiv {\sum }_{{n}_{a}}\left\vert {n}_{a}+1\right\rangle \left\langle {n}_{a}\right\vert$ is identified with ${e}^{{{\rm{i}}}{\delta }_{X}{\hat{p}}_{a}}$. Therefore, we can approximate ${\hat{p}}_{a}$ by $\frac{{\hat{S}}_{a}^{1/2}-{\hat{S}}_{a}^{-1/2}}{{{\rm{i}}}{\delta }_{X}}$ up to corrections of order *δ*_*X*_. Then,

17 $${\hat{p}}_{a}^{2} = \frac{2\cdot {{\bf{1}}}-{\hat{S}}_{a}-{\hat{S}}_{a}^{-1}}{{\delta }_{X}^{2}}=\frac{1}{{\delta }_{X}^{2}}{\sum }_{{n}_{a}=0}^{\Lambda -1}\left\{2\left\vert {n}_{a}\right\rangle \left\langle {n}_{a}\right\vert -\left\vert {n}_{a}+1\right\rangle \left\langle {n}_{a}\right\vert -\left\vert {n}_{a}\right\rangle \left\langle {n}_{a}+1\right\vert \right\}\,.$$

Because ${\sum }_{{n}_{a}}\left\vert {n}_{a}\right\rangle \left\langle {n}_{a}\right\vert$ is the identity, the nontrivial parts of ${\hat{p}}_{a}^{2}$ are ${\hat{S}}_{a}={\sum }_{{n}_{a}}\left\vert {n}_{a}+1\right\rangle \left\langle {n}_{a}\right\vert$ and ${\hat{S}}_{a}^{-1}$.

By applying the quantum Fourier transform, we can switch to the momentum eigenstates $\left\vert {\tilde{n}}_{a}\right\rangle$ (${\tilde{n}}_{a}=0,1,\cdots \,,\Lambda -1$):

18 $$\left\vert {\tilde{n}}_{a}\right\rangle =\frac{1}{\sqrt{\Lambda }}\sum\limits_{{n}_{a}}{e}^{2\pi {{\rm{i}}}{\tilde{n}}_{a}({n}_{a}+1/2)/\Lambda }\left\vert {n}_{a}\right\rangle \,.$$

The shift operator becomes diagonal in the momentum basis:

19 $$\hat{S}\left\vert {\tilde{n}}_{a}\right\rangle ={e}^{2\pi {{\rm{i}}}{\tilde{n}}_{a}/\Lambda }\left\vert {\tilde{n}}_{a}\right\rangle \,.$$

Therefore, ${\hat{p}}_{a}$ is diagonal, too:

20 $${\hat{p}}_{a}\left\vert {\tilde{n}}_{a}\right\rangle =\frac{2}{{\delta }_{X}}\sin \left(\frac{\pi {\tilde{n}}_{a}}{\Lambda }\right)\left\vert {\tilde{n}}_{a}\right\rangle \,.$$

Alternatively, we can define ${\hat{p}}_{a}$ as

21 $${\hat{p}}_{a}\left\vert {\tilde{n}}_{a}\right\rangle =\frac{2\pi }{{\delta }_{X}\Lambda }\left({\tilde{n}}_{a}+\frac{1}{2}\right)\left\vert {\tilde{n}}_{a}\right\rangle = \frac{\pi }{R}\left({\tilde{n}}_{a}+\frac{1}{2}\right)\left\vert {\tilde{n}}_{a}\right\rangle ,$$

restricting the range of ${\tilde{n}}_{a}$ from $-\frac{\Lambda }{2}$ to $+\frac{\Lambda }{2}-1$ instead of (20). Here, we used ${\tilde{n}}_{a}+1/2$ rather than ${\tilde{n}}_{a}$ to respect the symmetry under ${\hat{p}}_{a}\to -{\hat{p}}_{a}$. Associated with this, the Fourier transform is modified to

22 $$\left\vert {\tilde{n}}_{a}\right\rangle =\frac{1}{\sqrt{\Lambda }}\sum\limits_{{n}_{a}}{e}^{2\pi {{\rm{i}}}({\tilde{n}}_{a}+1/2)({n}_{a}+1/2)/\Lambda }\left\vert {n}_{a}\right\rangle \,.$$

With this option, the kinetic term is nonlocal in the coordinate basis. These two options are the same up to truncation effects.

Note that the Fourier transform can be performed for each boson in parallel, and hence the depth of the circuit depends only on the truncation level *Λ* and *not* on the number of bosons. Therefore, if we can perform a Fourier transform on a one-boson system, like the anharmonic oscillator (3), in principle, we only have to add more qubits that can describe more bosons and add the same circuits for the other bosons.

### Scalar quantum field theory

An important example of quantum field theory is a scalar *ϕ*^4^ theory in 3 + 1 spacetime dimensions. Despite its simplicity, this theory contains many important features of quantum field theory, and it is often used to demonstrate new concepts or new techniques. See e.g., the famous textbooks by Peskin and Schröder^41^ and by Fradkin^42^. Naturally, the seminal paper on quantum computing by ref. ^43^ studies this theory, too.

We regularize this theory on a cubic lattice with equal lattice spacing *a* in all three directions. Following the notations in ref. ^39^, we write the lattice Hamiltonian as

23 $$\hat{H}=\sum\limits_{\overrightarrow{n}}\left(\frac{1}{2}{\hat{\pi }}_{\overrightarrow{n}}^{2}+\frac{1}{2}{\sum }_{j=1}^{3}{\left({\hat{\phi }}_{\overrightarrow{n}+\hat{j}}-{\hat{\phi }}_{\overrightarrow{n}}\right)}^{2}+\frac{{m}^{2}}{2}{\hat{\phi }}_{\overrightarrow{n}}^{2}+\frac{\lambda }{4}{\hat{\phi }}_{\overrightarrow{n}}^{4}\right)\,.$$

The scalar field $\hat{\phi }$ and its conjugate momentum $\hat{\pi }$ are dimensionless. They correspond to fields in the continuum theory according to $\hat{\phi }=a{\hat{\phi }}_{{{\rm{cont.}}}}$ and $\hat{\pi }={a}^{2}{\hat{\pi }}_{{{\rm{cont.}}}}$ (where *a* is the dimensionfull lattice spacing). The Hamiltonian and the mass parameter are also made dimensionless, i.e., $\hat{H}=a\times {\hat{H}}_{{{\rm{cont.}}}}$, *m* = *a* × *m*_cont._. The lattice sites are labeled by $\overrightarrow{n}\in {{\mathbb{Z}}}^{d}$; and $\hat{j}$ is the unit vector along the *j*th dimension of the spatial lattice (*j* = 1, 2, 3).

The canonical commutation relation is imposed, i.e.,

24 $$[{\hat{\phi }}_{\overrightarrow{n}},{\hat{\pi }}_{{\overrightarrow{n}}^{{\prime} }}]={{\rm{i}}}{\delta }_{\overrightarrow{n},{\overrightarrow{n}}^{{\prime} }}\,.$$

These operators $\hat{\phi }$ and $\hat{\pi }$ are the same as $\hat{x}$ and $\hat{p}$ in the previous sections, and the Hamiltonian takes the universal form (1).

As a minor comment on terminology, we note that the quadratic term in $\hat{\phi }$ in the lattice Hamiltonian (23) corresponds to the spatial derivative term ${({\partial }_{j}\hat{\phi })}^{2}$ in the continuum theory, which is usually called a kinetic term. However, in the context of the universal form (1), we regard it as a quadratic part of the potential $V(\hat{x})$, because $\hat{\phi }$ is playing the role of a (bosonic) coordinate.

Next, we define the Hilbert space describing the lattice system. A convenient way to define the Hilbert space is to use coordinate eigenstates $\left\vert \phi \right\rangle$ that satisfy ${\hat{\phi }}_{\overrightarrow{n}}\left\vert \phi \right\rangle ={\phi }_{\overrightarrow{n}}\left\vert \phi \right\rangle$

25 $${{\mathcal{H}}}=\left\{\left\vert \Psi \right\rangle \equiv \int{{{\rm{d}}}}^{{V}_{{{\rm{lattice}}}}}\phi \,\Psi (\phi )\left\vert \phi \right\rangle | \int{{{\rm{d}}}}^{{V}_{{{\rm{lattice}}}}}\phi \,| \Psi (\phi ){| }^{2} < \infty \right\}\,,$$

where *V*_lattice_ is the lattice volume (the number of lattice points). We can also use the momentum eigenstates $\left\vert \pi \right\rangle$ that satisfy ${\hat{\pi }}_{\overrightarrow{n}}\left\vert \pi \right\rangle ={\pi }_{\overrightarrow{n}}\left\vert \pi \right\rangle$

26 $${{\mathcal{H}}}=\left\{\left\vert \Psi \right\rangle \equiv \int{{{\rm{d}}}}^{{V}_{{{\rm{lattice}}}}}\pi \,\tilde{\Psi }(\pi )\left\vert \pi \right\rangle \left\vert \right.\int{{{\rm{d}}}}^{{V}_{{{\rm{lattice}}}}}\pi \,| \tilde{\Psi }(\pi ){| }^{2} < \infty \right\}\,.$$

Wave functions *Ψ*(*ϕ*) and $\tilde{\Psi }(\pi )$ are related by the Fourier transform. Note that

27 $$\left\langle \pi \right\vert \left\vert \phi \right\rangle =\exp \left(-{{\rm{i}}}\sum\limits_{\overrightarrow{n}}{\pi }_{\overrightarrow{n}}{\phi }_{\overrightarrow{n}}\right)\,.$$

We often use a notation

28 $${{\mathcal{H}}}={{\rm{Span}}}\left\{\left\vert \phi \right\rangle | \phi \in {{\mathbb{R}}}^{{V}_{{{\rm{lattice}}}}}\right\}={{\rm{Span}}}\left\{\left\vert \pi \right\rangle | \pi \in {{\mathbb{R}}}^{{V}_{{{\rm{lattice}}}}}\right\}$$

assuming the square-integrability condition.

We assign *Q* qubits to each bosonic degree of freedom. Then, the number of qubits needed to describe the Hilbert space is *QV*_lattice_.

### Matrix models

Now we turn our attention to the SU(*N*) bosonic *d*-matrix model, and again we realize that we are dealing with a Hamiltonian of the form (1). The Lagrangian is

29 $$L={{\rm{Tr}}}\left(\frac{1}{2}{({D}_{t}{X}_{I})}^{2}-\frac{{g}^{2}}{4}{[{X}_{I},{X}_{J}]}^{2}\right)\,,$$

where *X*_*I*=1,2.⋯ ,*d*_ are *N* × *N* Hermitian matrices and *D*_*t*_*X*_*I*_ is the gauge covariant derivative defined by *D*_*t*_*X*_*I*_ = ∂_*t*_*X*_*I*_ − *ig*[*A*_*t*_, *X*_*I*_]. The integration of the gauge field *A*_*t*_ leads to the Gauss-law constraint.

We can either impose or not impose a traceless condition on *X*_*I*_. Both options are explained below. There is no difference in physics because the trace part is free and decoupled from the rest.

Below, we confirm that this model belongs to a class of theories whose Hamiltonians take the simple form in (1). Let us first consider the case with the traceless condition. To have real expansion coefficients, we introduce SU(*N*) generators *τ*_*α*_, where the adjoint index *α* runs from 1 to *N*^2^ − 1, that are normalized as ${{\rm{Tr}}}({\tau }_{\alpha }{\tau }_{\beta })={\delta }_{\alpha \beta }$. Then the matrix elements are written as

30 $${X}_{I,ij}={\sum }_{\alpha =1}^{{N}^{2}-1}{X}_{I}^{\alpha }{\tau }_{\alpha ,ij}\,\qquad {X}_{I}^{\alpha }\in {\mathbb{R}}\,.$$

By using the structure constant ${f}_{\alpha \beta }^{\gamma }$, which are related to the generators through $[{\tau }_{\alpha },{\tau }_{\beta }]={{\rm{i}}}{\sum }_{\gamma }{f}_{\alpha \beta }^{\gamma }{\tau }_{\gamma }$, we have $[{X}_{I},{X}_{J}]={{\rm{i}}}{\sum }_{\alpha ,\beta ,\gamma }{f}_{\alpha \beta }^{\gamma }{X}_{I}^{\alpha }{X}_{J}^{\beta }{\tau }_{\gamma }$. See Supplementary Note 1 for an explicit construction of the generators.

The corresponding Hamiltonian is

31 $$\hat{H}={{\rm{Tr}}}\left(\frac{1}{2}{\hat{P}}_{I}^{2}-\frac{{g}^{2}}{4}{[{\hat{X}}_{I},{\hat{X}}_{J}]}^{2}\right)\,.$$

Here $[{\hat{X}}_{I},{\hat{X}}_{J}]$ denotes the commutator of *N* × *N* matrices, i.e.,

$${[{\hat{X}}_{I},{\hat{X}}_{J}]}_{ij}	={({\hat{X}}_{I}{\hat{X}}_{J})}_{ij}-{({\hat{X}}_{J}{\hat{X}}_{I})}_{ij}\\ 	={\sum }_{k=1}^{N}\left({\hat{X}}_{I,ik}{\hat{X}}_{J,kj}-{\hat{X}}_{J,ik}{\hat{X}}_{I,kj}\right)\,.$$

The symbol “${{\rm{Tr}}}$” means the trace as an *N* × *N* matrix. When indices are written explicitly in the commutator, it means a commutator as operator,

$$[{\hat{X}}_{I,ij},{\hat{X}}_{J,kl}]={\hat{X}}_{I,ij}{\hat{X}}_{J,kl}-{\hat{X}}_{J,kl}{\hat{X}}_{I,ij}\,.$$

Note also that ${({\hat{X}}_{I,ij})}^{{\dagger} }={\hat{X}}_{I,ji}$ and ${({\hat{P}}_{I,ij})}^{{\dagger} }={\hat{P}}_{I,ji}$. We can introduce the operators with the adjoint index *α* as

32 $${\hat{X}}_{I,ij}=\sum\limits_{\alpha }{\hat{X}}_{I}^{\alpha }{\tau }_{\alpha ,ij}\,,\qquad {\hat{P}}_{I,ij}=\sum\limits_{\alpha }{\hat{P}}_{I}^{\alpha }{\tau }_{\alpha ,ij}\,.$$

${\hat{X}}_{I}^{\alpha }$ and ${\hat{P}}_{I}^{\alpha }$ are self-adjoint, i.e., ${({\hat{X}}_{I}^{\alpha })}^{{\dagger} }={\hat{X}}_{I}^{\alpha }$, ${({\hat{P}}_{I}^{\alpha })}^{{\dagger} }={\hat{P}}_{I}^{\alpha }$, and they satisfy the canonical commutation relations

33 $$[{\hat{X}}_{I}^{\alpha },{\hat{P}}_{J}^{\beta }]={{\rm{i}}}{\delta }_{IJ}{\delta }_{\alpha \beta }\,,\qquad [{\hat{X}}_{I}^{\alpha },{\hat{X}}_{J}^{\beta }]=[{\hat{P}}_{I}^{\alpha },{\hat{P}}_{J}^{\beta }]=0\,,$$

where these commutators are understood as operator commutators, e.g.,

$$[{\hat{X}}_{I}^{\alpha },{\hat{P}}_{J}^{\beta }]={\hat{X}}_{I}^{\alpha }{\hat{P}}_{J}^{\beta }-{\hat{P}}_{J}^{\beta }{\hat{X}}_{I}^{\alpha }\,.$$

By using ${\hat{X}}_{I}^{\alpha }$ and ${\hat{P}}_{I}^{\alpha }$, the Hamiltonian (31) can be written in the simple form (1) with *d*(*N*^2^ − 1) real bosonic degrees of freedom.

In contrast, let us now consider the case without the traceless condition. If we do not impose the traceless condition, we do not need to use generators. We could simply use the diagonal entries ${\hat{X}}_{I,ii}$, which are real, and the real and imaginary parts of the off-diagonal entries,

34 $${\hat{X}}_{I,ij}^{{{\rm{(R)}}}}\equiv \frac{1}{\sqrt{2}}({\hat{X}}_{I,ij}+{\hat{X}}_{I,ji})\,\qquad {\hat{X}}_{I,ij}^{{{\rm{(I)}}}}\equiv \frac{-{{\rm{i}}}}{\sqrt{2}}({\hat{X}}_{I,ij}-{\hat{X}}_{I,ji})$$

as real bosonic operators with canonical normalization.

The trace part is free and decouples from the SU(*N*) sector under time evolution, if the initial momentum of the trace part is zero, it just stays zero. To stabilize the trace part regardless of the initial condition, we can add a mass term proportional to ${({{\rm{Tr}}}{\hat{X}}_{I})}^{2}$.

Next, we define the Hilbert space associated with this system. In the case with the traceless condition, a convenient way to define the Hilbert space is to use coordinate eigenstates $\left\vert X\right\rangle$ that satisfy ${\hat{X}}_{I}^{\alpha }\left\vert X\right\rangle ={X}_{I}^{\alpha }\left\vert X\right\rangle$

35 $${{{\mathcal{H}}}}_{{{\rm{ext}}}}=\left\{\left\vert \Psi \right\rangle \equiv \int{{{\rm{d}}}}^{d({N}^{2}-1)}X\,\Psi (X)\left\vert X\right\rangle \left| \int{{{\rm{d}}}}^{d({N}^{2}-1)} \right. X\,| \Psi (X){| }^{2} < \infty \right\}\,.$$

Here the subscripts ext indicate that ${{{\mathcal{H}}}}_{{{\rm{ext}}}}$ is the *extended* Hilbert space that contains SU(*N*) non-singlets. We can also use the momentum eigenstates $\left\vert P\right\rangle$ that satisfy ${\hat{P}}_{I}^{\alpha }\left\vert P\right\rangle ={P}_{I}^{\alpha }\left\vert P\right\rangle$

36 $${{{\mathcal{H}}}}_{{{\rm{ext}}}}=\left\{\left\vert \Psi \right\rangle \equiv \int{{{\rm{d}}}}^{d({N}^{2}-1)}P\,\tilde{\Psi }(P)\left\vert P\right\rangle \left| \right.\int{{{\rm{d}}}}^{d({N}^{2}-1)}P\,| \tilde{\Psi }(P){| }^{2} < \infty \right\}\,.$$

Wave functions *Ψ*(*X*) and $\tilde{\Psi }(P)$ are related by the Fourier transform. Note that

37 $$\left\langle P\right\vert \left\vert X\right\rangle =\exp \left(-{{\rm{i}}}\sum\limits_{I,\alpha }{P}_{I}^{\alpha }{X}_{I}^{\alpha }\right)=\exp \left(-{{\rm{i}}}\sum\limits_{I}{{\rm{Tr}}}({P}_{I}{X}_{I})\right)\,.$$

We often use the notation

38 $${{{\mathcal{H}}}}_{{{\rm{ext}}}}={{\rm{Span}}}\left\{\left\vert X\right\rangle | X\in {{\mathbb{R}}}^{d({N}^{2}-1)}\right\}={{\rm{Span}}}\left\{\left\vert P\right\rangle | P\in {{\mathbb{R}}}^{d({N}^{2}-1)}\right\}$$

assuming the square-integrability condition.

Under the SU(*N*) gauge transformation, these states transform according to

39 $$\left\vert X\right\rangle \to \left\vert {\Omega }^{-1}X\Omega \right\rangle \,,\qquad \left\vert P\right\rangle \to \left\vert {\Omega }^{-1}P\Omega \right\rangle \,,$$

while the operators transform as

40 $$\begin{array}{rcl}{\hat{X}}_{I,ij}&\to &{(\Omega {\hat{X}}_{I}{\Omega }^{-1})}_{ij}=\sum\limits_{k,l}{\Omega }_{ik}{\hat{X}}_{I,kl}{\Omega }_{lj}^{-1}\,,\\ {\hat{P}}_{I,ij}&\to &{(\Omega {\hat{P}}_{I}{\Omega }^{-1})}_{ij}=\sum\limits_{k,l}{\Omega }_{ik}{\hat{P}}_{I,kl}{\Omega }_{lj}^{-1}\,.\end{array}$$

Gauge-invariant states span a subspace of ${{{\mathcal{H}}}}_{{{\rm{ext}}}}$ which we denote by ${{{\mathcal{H}}}}_{{{\rm{inv}}}}$. To take into account the gauge-singlet constraint, one can either restrict the Hilbert space to ${{{\mathcal{H}}}}_{{{\rm{inv}}}}$, or one can identify the states in ${{{\mathcal{H}}}}_{{{\rm{ext}}}}$ that transform to each other under SU(*N*) transformations. ${{{\mathcal{H}}}}_{{{\rm{ext}}}}$ admits the truncation scheme discussed in Sec. Basic idea.

We assign *Q* qubits to each bosonic degree of freedom. Then, the number of qubits needed to describe the Hilbert space is *d*(*N*^2^ − 1)*Q*.

If the traceless condition is not imposed, we can repeat the same construction simply by replacing ${{\mathbb{R}}}^{d({N}^{2}-1)}$ with ${{\mathbb{R}}}^{d{N}^{2}}$.

Next, let us take a closer look at the Hamiltonian (31). This analysis will be useful for estimating the computational cost discussed in later sections.

When the traceless condition is imposed, the kinetic term takes the form

41 $$\frac{1}{2}{\sum }_{I=1}^{d}{{\rm{Tr}}}{\hat{P}}_{I}^{2}=\frac{1}{2}{\sum }_{I=1}^{d}{\sum }_{\alpha =1}^{{N}^{2}-1}{({\hat{P}}_{I}^{\alpha })}^{2}\,.$$

Therefore, there are *d*(*N*^2^ − 1) terms in the kinetic part. When the traceless condition is not imposed, there are instead *dN*^2^ terms:

42 $$\frac{1}{2}{\sum }_{I=1}^{d}{{\rm{Tr}}}{\hat{P}}_{I}^{2}=\frac{1}{2}{\sum }_{I=1}^{d}\left[\sum\limits_{i < j}\left({({\hat{P}}_{I,ij}^{{{\rm{(R)}}}})}^{2}+{({\hat{P}}_{I,ij}^{{{\rm{(I)}}}})}^{2}\right)+\sum\limits_{i}{({\hat{P}}_{I,ii})}^{2}\right].$$

In either case, the kinetic term takes the standard form ${\sum }_{a}{\hat{p}}_{a}^{2}/2$.

A simple way to treat the kinetic term is to use the quantum Fourier transform and change the basis to the momentum basis. The operation of the gates can be completely parallelized because ${\hat{p}}_{a}^{2}$ is diagonal in the momentum basis.

Next, we turn to the potential term. When a traceless condition is imposed, we can write the potential term as

43 $${{\rm{Tr}}}{[{\hat{X}}_{I},{\hat{X}}_{J}]}^{2}	= \sum\limits_{\gamma }\left({{\rm{i}}}\sum\limits_{\alpha \beta }{\hat{X}}_{I}^{\alpha }{\hat{X}}_{J}^{\beta }{f}_{\alpha \beta }^{\gamma }\right)\left({{\rm{i}}}\sum\limits_{{\alpha }^{{\prime} },{\beta }^{{\prime} }}{\hat{X}}_{I}^{{\alpha }^{{\prime} }}{\hat{X}}_{J}^{{\beta }^{{\prime} }}{f}_{{\alpha }^{{\prime} }{\beta }^{{\prime} }}^{\gamma }\right)\\ 	 \equiv \sum\limits_{\alpha ,\beta ,{\alpha }^{{\prime} },{\beta }^{{\prime} }}{C}^{\alpha \beta {\alpha }^{{\prime} }{\beta }^{{\prime} }}{\hat{X}}_{I}^{\alpha }{\hat{X}}_{J}^{\beta }{\hat{X}}_{I}^{{\alpha }^{{\prime} }}{\hat{X}}_{J}^{{\beta }^{{\prime} }}\,,$$

where

44 $${C}^{\alpha \beta {\alpha }^{{\prime} }{\beta }^{{\prime} }}\equiv -\sum\limits_{\gamma }{f}_{\alpha \beta }^{\gamma }{f}_{{\alpha }^{{\prime} }{\beta }^{{\prime} }\gamma }\,.$$

There are order *N*^4^ nonzero components of *C*. The number of combinations for *I*, *J* is *d*(*d* − 1)/2. Therefore, the number of quartic interaction terms in (43) scales as *d*(*d* − 1)*N*^4^. It is straightforward to write ${C}^{\alpha \beta {\alpha }^{{\prime} }{\beta }^{{\prime} }}$ for a given choice of generators using any computer algebra system.

When we do not impose the traceless condition, we go back to (31) and look at the commutator term. We can examine the terms ${{\rm{Tr}}}({X}_{I}{X}_{J}{X}_{I}{X}_{J})$ and ${{\rm{Tr}}}({X}_{I}{X}_{I}{X}_{J}{X}_{J})$ separately. As example, let us see the former, which can be written as

45 $$\sum\limits_{i,j,k,l}{X}_{I,ij}{X}_{J,jk}{X}_{I,kl}{X}_{J,li}\,.$$

For the counting to the leading order in *N*, we can assume that *i*, *j*, *k*, *l* are all different. Hence, there are *N*^4^ terms in the sum. In this way, we can see that the number of terms in the potential term scales as *d*(*d* − 1)*N*^4^.

Finally, to impose the singlet constraint, we introduce a penalty term. By using the structure constant *f*_*αβγ*_ that is totally antisymmetric and related to the generators by [*τ*_*α*_, *τ*_*β*_] = i*f*_*αβγ*_*τ*_*γ*_, generators of gauge transformations can be written as

46 $${\hat{G}}_{\alpha }={{\rm{i}}}\sum\limits_{I,\beta ,\gamma }{f}_{\alpha \beta \gamma }{\hat{X}}_{I,\beta }{\hat{P}}_{I,\gamma }\,.$$

Note that there is no ambiguity in the operator ordering on the right-hand side because *f*_*αβγ*_ = 0 if *β* = *γ*.

One way to forbid SU(*N*) non-singlet states explicitly is to add to the Hamiltonian a penalty term $c{\sum }_{\alpha }{\hat{G}}_{\alpha }^{2}$ with a large positive coefficient *c*^44–46^. In this paper, we do not consider this option. If the Hamiltonian time evolution is precise, then it respects SU(*N*) invariance, and hence the gauge-invariant sector of the Hilbert space will not be left.

### Orbifold lattice

Next, we study (*d* + 1)-dimensional SU(*N*) Yang–Mills theory defined on the orbifold lattice^36^. By default, orbifold lattice theory has U(*N*) gauge fields rather than SU(*N*). For pure Yang–Mills theory, the U(1) part decouples and the SU(*N*) part is not affected. See refs. ^36,37^ for the removal of the U(1) part in QCD. The present discussion focuses on *d* = 2 and *d* = 3, but we keep *d* arbitrary as we can equally easily treat larger dimensions. The number of lattice sites is *L*^*d*^ = *V*_lattice_, and periodic boundary conditions are assumed.

If you already know the orbifold lattice construction, an easy way to understand why its use simplifies simulations is to notice that the orbifold lattice is obtained from the SU(*NL*^*d*^) 2*d*-matrix model via the orbifold projection^47^. Therefore, if we can simulate the matrix model with the Hamiltonian discussed in the previous section, we can also simulate the orbifold lattice gauge theory.

The orbifold lattice Hamiltonian can be written in terms of the complex link variables ${Z}_{j,\overrightarrow{n}}$ and their canonical conjugates ${P}_{j,\overrightarrow{n}}$. For *d* = 3, the Hamiltonian is

47 $$\begin{array}{l}\hat{H}=\sum\limits_{\overrightarrow{n}}{{\rm{Tr}}}\left({\sum }_{j=1}^{3}{\hat{P}}_{j,\overrightarrow{n}}{\hat{\bar{P}}}_{j,\overrightarrow{n}}+\frac{{g}_{{{\rm{4d}}}}^{2}}{2{a}^{3}}{\left\vert {\sum }_{j = 1}^{3}\left({\hat{Z}}_{j,\overrightarrow{n}}{\hat{\bar{Z}}}_{j,\overrightarrow{n}}-{\hat{\bar{Z}}}_{j,\overrightarrow{n}-\hat{j}}{\hat{Z}}_{j,\overrightarrow{n}-\hat{j}}\right)\right\vert }^{2}\right.\\ \left.+\frac{2{g}_{{{\rm{4d}}}}^{2}}{{a}^{3}}\sum\limits_{j < k}{\left\vert {\hat{Z}}_{j,\overrightarrow{n}}{\hat{Z}}_{k,\overrightarrow{n}+\hat{j}}-{\hat{Z}}_{k,\overrightarrow{n}}{\hat{Z}}_{j,\overrightarrow{n}+\hat{k}}\right\vert }^{2}\right)+\Delta \hat{H}\,.\end{array}$$

For *d* = 2, the same expression applies with the replacement ${g}_{{{\rm{4d}}}}^{2}\to a{g}_{{{\rm{3d}}}}^{2}$, where *a* is the lattice spacing. The additional contribution is

48 $$\Delta \hat{H} \equiv 	 \frac{{m}^{2}{g}_{{{\rm{4d}}}}^{2}}{2a}\sum\limits_{\overrightarrow{n}}{\sum }_{j=1}^{3}{{\rm{Tr}}}{\left\vert {\hat{Z}}_{j,\overrightarrow{n}}{\hat{\bar{Z}}}_{j,\overrightarrow{n}}-\frac{a}{2{g}_{{{\rm{4d}}}}^{2}}\right\vert }^{2} \\ 	 +\frac{N{\mu }^{2}{g}_{{{\rm{4d}}}}^{2}}{2a}\sum\limits_{\overrightarrow{n}}{\sum }_{j=1}^{3}{\left\vert \frac{1}{N}{{\rm{Tr}}}({\hat{Z}}_{j,\overrightarrow{n}}{\hat{\bar{Z}}}_{j,\overrightarrow{n}})-\frac{a}{2{g}_{{{\rm{4d}}}}^{2}}\right\vert }^{2}. $$

Note that ${\bar{Z}}_{j,\overrightarrow{n}}$ and ${\bar{P}}_{j,\overrightarrow{n}}$ stand for Hermitian conjugates of *N* × *N* matrices, i.e.,

49 $${\bar{Z}}_{j,\overrightarrow{n};ab}={({Z}_{j,\overrightarrow{n};ba})}^{* }\,,\qquad {\bar{P}}_{j,\overrightarrow{n};ab}={({P}_{j,\overrightarrow{n};ba})}^{* }\,.$$

We do not use dagger † because we save it for conjugate operators acting on the Hilbert space. For the operators this implies

50 $${\hat{\bar{Z}}}_{j,\overrightarrow{n};ab}={\left({\hat{Z}}_{j,\overrightarrow{n};ba}\right)}^{{\dagger} }\,,\qquad {\hat{\bar{P}}}_{j,\overrightarrow{n};ab}={\left({\hat{P}}_{j,\overrightarrow{n};ba}\right)}^{{\dagger} }\,,$$

The canonical commutation relation is

51 $$[{\hat{Z}}_{j,\overrightarrow{n};ab},{\hat{\bar{P}}}_{k,{\overrightarrow{n}}^{{\prime} };cd}]=[{\hat{Z}}_{j,\overrightarrow{n};ab},{\left({\hat{P}}_{k,{\overrightarrow{n}}^{{\prime} };dc}\right)}^{{\dagger} }]={{\rm{i}}}{\delta }_{jk}{\delta }_{\overrightarrow{n}{\overrightarrow{n}}^{{\prime} }}{\delta }_{ad}{\delta }_{bc}\,.$$

We denote the real and imaginary parts by the superscripts (R) and (I), respectively, to rewrite the variables and commutation relations as

52 $${\hat{Z}}_{j,\overrightarrow{n};ab}=\frac{{\hat{Z}}_{j,\overrightarrow{n};ab}^{{{\rm{(R)}}}}+{{\rm{i}}}{\hat{Z}}_{j,\overrightarrow{n};ab}^{{{\rm{(I)}}}}}{\sqrt{2}}\,,\qquad {\hat{P}}_{j,\overrightarrow{n};ab}=\frac{{\hat{P}}_{j,\overrightarrow{n};ab}^{{{\rm{(R)}}}}+{{\rm{i}}}{\hat{P}}_{j,\overrightarrow{n};ab}^{{{\rm{(I)}}}}}{\sqrt{2}}\,.$$

The real and imaginary parts are taken to be self-adjoint, i.e.,

53 $${\left({\hat{Z}}_{j,\overrightarrow{n};ab}^{{{\rm{(R)}}}}\right)}^{{\dagger} }={\hat{Z}}_{j,\overrightarrow{n};ab}^{{{\rm{(R)}}}}\,,\qquad {\left({\hat{Z}}_{j,\overrightarrow{n};ab}^{{{\rm{(I)}}}}\right)}^{{\dagger} }={\hat{Z}}_{j,\overrightarrow{n};ab}^{{{\rm{(I)}}}}\,,$$

and the same for $\hat{P}$. Therefore, in terms of complex operators,

54 $${\hat{Z}}_{j,\overrightarrow{n};ab}^{{{\rm{(R)}}}}=\frac{{\hat{Z}}_{j,\overrightarrow{n};ab}+{\left({\hat{Z}}_{j,\overrightarrow{n};ab}\right)}^{{\dagger} }}{\sqrt{2}}=\frac{{\hat{Z}}_{j,\overrightarrow{n};ab}+{\hat{\bar{Z}}}_{j,\overrightarrow{n};ba}}{\sqrt{2}}\,,$$

and so on. The commutation relation is

55 $$[{\hat{Z}}_{j,\overrightarrow{n};ab}^{{{\rm{(R)}}}},{\hat{P}}_{k,{\overrightarrow{n}}^{{\prime} };cd}^{{{\rm{(R)}}}}]=[{\hat{Z}}_{j,\overrightarrow{n};ab}^{{{\rm{(I)}}}},{\hat{P}}_{k,{\overrightarrow{n}}^{{\prime} };cd}^{{{\rm{(I)}}}}]={{\rm{i}}}{\delta }_{jk}{\delta }_{\overrightarrow{n}{\overrightarrow{n}}^{{\prime} }}{\delta }_{ac}{\delta }_{bd}\,.$$

In terms of ${\hat{Z}}^{{{\rm{(R)}}}}$ and ${\hat{Z}}^{{{\rm{(I)}}}}$, the Hamiltonian reduces to the form (1).

So far, the Hamiltonian is merely a quiver matrix model. (See e.g., ref. ^48^ for a review on quivers in the context of quantum field theory and string theory.) A crucial step is to generate a lattice by using dimensional deconstruction^49^. To see how Yang–Mills theory is obtained from this Hamiltonian, we write ${Z}_{j,\overrightarrow{n}}$ as

56 $${Z}_{j,\overrightarrow{n}}=\sqrt{\frac{a}{2{g}_{{{\rm{4d}}}}^{2}}}{W}_{j,\overrightarrow{n}}{U}_{j,\overrightarrow{n}}\,$$

where ${U}_{j,\overrightarrow{n}}$ is unitary and ${W}_{j,\overrightarrow{n}}\equiv \sqrt{\frac{2{d}_{{{\rm{4d}}}}^{2}}{a}}\sqrt{{Z}_{j,\overrightarrow{n}}{Z}_{j,\overrightarrow{n}}^{{\dagger} }}$ is a positive-definite Hermitian matrix. By writing ${U}_{j,\overrightarrow{n}}$ and ${W}_{j,\overrightarrow{n}}$ as ${U}_{j,\overrightarrow{n}}=\exp \left({{\rm{i}}}a{g}_{{{\rm{4d}}}}{A}_{j,\overrightarrow{n}}\right)$ and ${W}_{j,\overrightarrow{n}}=\exp \left(a{g}_{{{\rm{4d}}}}{\phi }_{j,\overrightarrow{n}}\right)$, respectively, we can interpret *A*_*j*_ and *ϕ*_*j*_ as the gauge fields and adjoint scalars^47^. To justify this interpretation, we can stabilize scalars by introducing a large mass in the additional term $\Delta \hat{H}$. Nonzero vacuum expectation values of scalars effectively shift the lattice spacing. Optionally, one could add a quadratic term ${{\rm{Tr}}}({\hat{Z}}_{j,\overrightarrow{n}}{\hat{\bar{Z}}}_{j,\overrightarrow{n}})$ to control the vacuum expectation value of scalars without introducing an extremely large bare mass. Then, we obtain Yang–Mills theory coupled to scalar fields. Indeed, if we turn off the scalars (i.e., choosing *W* to be the identity), the term ${{\rm{Tr}}}{\left\vert {\sum }_{j = 1}^{3}\left({\hat{Z}}_{j,\overrightarrow{n}}{\hat{\bar{Z}}}_{j,\overrightarrow{n}}-{\hat{\bar{Z}}}_{j,\overrightarrow{n}-\hat{j}}{\hat{Z}}_{j,\overrightarrow{n}-\hat{j}}\right)\right\vert }^{2}$ in (47) becomes zero while the term, ${\sum }_{j < k}{{\rm{Tr}}}{\left\vert {\hat{Z}}_{j,\overrightarrow{n}}{\hat{Z}}_{k,\overrightarrow{n}+\hat{j}}-{\hat{Z}}_{k,\overrightarrow{n}}{\hat{Z}}_{j,\overrightarrow{n}+\hat{k}}\right\vert }^{2}$ gives the plaquette term, which leads to the magnetic term of Yang–Mills theory. Focusing on the leading order in *ϕ*, the first term gives ${{\rm{Tr}}}{({\sum }_{j}{D}_{j}{\phi }_{j})}^{2}$ while the second term gives ${\sum }_{j < k}{{\rm{Tr}}}{({D}_{j}{\phi }_{k}-{D}_{k}{\phi }_{j})}^{2}$, which sum up to ${\sum }_{j,k}{{\rm{Tr}}}{({D}_{j}{\phi }_{k})}^{2}$ up to total derivatives. The quartic interaction ${{\rm{Tr}}}{[{\phi }_{j},{\phi }_{k}]}^{2}$ appears as well. See refs. ^36,37^ for details. At low energy, large mass scalars decouple and pure Yang–Mills theory is obtained. The mass of scalars can be as large as the lattice cutoff scale.

We now define the Hilbert space used in this formulation. SU(*N*) gauge transformations are defined by ${Z}_{j,\overrightarrow{n}}\to {\Omega }_{\overrightarrow{n}}^{-1}{Z}_{j,\overrightarrow{n}}{\Omega }_{\overrightarrow{n}+\hat{j}}$, which is equivalent to ${U}_{j,\overrightarrow{n}}\to {\Omega }_{\overrightarrow{n}}^{-1}{U}_{j,\overrightarrow{n}}{\Omega }_{\overrightarrow{n}+\hat{j}}$ and ${W}_{j,\overrightarrow{n}}\to {\Omega }_{\overrightarrow{n}}^{-1}{W}_{j,\overrightarrow{n}}{\Omega }_{\overrightarrow{n}}$. We use the extended Hilbert space ${{{\mathcal{H}}}}_{{{\rm{ext}}}}$, which can be defined by using the coordinate eigenstates $\left\vert Z\right\rangle$ that satisfy ${\hat{Z}}_{j,\overrightarrow{n}}\left\vert Z\right\rangle ={Z}_{j,\overrightarrow{n}}\left\vert Z\right\rangle$ as

57 $${{{\mathcal{H}}}}_{{{\rm{ext}}}}=\left\{\left\vert \Psi \right\rangle \equiv \int{{{\rm{d}}}}^{2d{N}^{2}{V}_{{{\rm{lattice}}}}}Z\,\Psi (Z)\left\vert Z\right\rangle \left| \int{{{\rm{d}}}}^{2d{N}^{2}{V}_{{{\rm{lattice}}}}} \right. Z\,| \Psi (Z){| }^{2} < \infty \right\}\,.$$

We can also use the momentum eigenstates $\left\vert P\right\rangle$ that satisfy ${\hat{P}}_{j,\overrightarrow{n}}\left\vert P\right\rangle ={P}_{j,\overrightarrow{n}}\left\vert P\right\rangle$

58 $${{{\mathcal{H}}}}_{{{\rm{ext}}}}=\left\{\left\vert \Psi \right\rangle \equiv \int{{{\rm{d}}}}^{2d{N}^{2}{V}_{{{\rm{lattice}}}}}P\,\tilde{\Psi }(P)\left\vert P\right\rangle \left| \int{{{\rm{d}}}}^{2d{N}^{2}{V}_{{{\rm{lattice}}}}} \right. P\,| \tilde{\Psi }(P){| }^{2} < \infty \right\}.$$

Wave functions *Ψ*(*Z*) and $\tilde{\Psi }(P)$ are related by the Fourier transform. Under the SU(*N*) gauge transformation, these states transform as

59 $$\left\vert Z\right\rangle \to \left\vert {\Omega }^{-1}Z\Omega \right\rangle \,,\qquad \left\vert P\right\rangle \to \left\vert {\Omega }^{-1}P\Omega \right\rangle \,,$$

while the operators transform as

60 $$\begin{array}{rcl}{\hat{Z}}_{j,\overrightarrow{n};ab}&\to &{({\Omega }_{\overrightarrow{n}}{\hat{Z}}_{j,\overrightarrow{n}}{\Omega }_{\overrightarrow{n}+\hat{j}}^{-1})}_{ab}=\sum_{c,d}{\Omega }_{\overrightarrow{n};ac}{\hat{Z}}_{j,\overrightarrow{n};cd}{\Omega }_{\overrightarrow{n}+\hat{j};db}^{-1}\,,\\ {\hat{P}}_{j,\overrightarrow{n};ab}&\to &{({\Omega }_{\overrightarrow{n}}{\hat{P}}_{j,\overrightarrow{n}}{\Omega }_{\overrightarrow{n}+\hat{j}}^{-1})}_{ab}=\sum\limits_{c,d}{\Omega }_{\overrightarrow{n};ac}{\hat{P}}_{j,\overrightarrow{n};cd}{\Omega }_{\overrightarrow{n}+\hat{j};db}^{-1}\,.\end{array}$$

Gauge-invariant states span a subspace of ${{{\mathcal{H}}}}_{{{\rm{ext}}}}$ which we denote by ${{{\mathcal{H}}}}_{{{\rm{inv}}}}$.

In this paper, we do not impose an SU(*N*)-singlet constraint on the Hilbert space. There are *dV*_lattice_ links and each link carries 2*N*^2^ real bosonic degrees of freedom. Therefore, there are 2*N*^2^*dV*_lattice_ real bosonic degrees of freedom in total. We assign *Q* qubits to each of them, such that the number of qubits needed to describe the Hilbert space is 2*N*^2^*dV*_lattice_*Q*. Next, we take a closer look at the Hamiltonian. This analysis serves as a preparation for the cost estimate presented in later sections; readers primarily interested in the overall scaling may skip the following details. By construction, the kinetic term is

61 $$\hat{H}=\frac{1}{2}\sum\limits_{\overrightarrow{n}}{\sum }_{j=1}^{3}{\sum }_{a,b=1}^{N}\left({({\hat{P}}_{j,\overrightarrow{n};ab}^{{{\rm{(R)}}}})}^{2}+{({\hat{P}}_{j,\overrightarrow{n};ab}^{{{\rm{(I)}}}})}^{2}\right)\,,$$

which is the same as the standard form in (1).

There are 2*N*^2^*dV*_lattice_ terms, which can be easily treated by using a quantum Fourier transform and going to the momentum basis.

We then turn to the potential term in the Hamiltonian, which can be written as

62 $$\begin{array}{l}\frac{{g}_{{{\rm{4d}}}}^{2}}{{a}^{3}}\sum\limits_{\overrightarrow{n}}{{\rm{Tr}}}\sum\limits_{j}\left({\hat{Z}}_{j,\overrightarrow{n}}{\hat{\bar{Z}}}_{j,\overrightarrow{n}}{\hat{Z}}_{j,\overrightarrow{n}}{\hat{\bar{Z}}}_{j,\overrightarrow{n}}-{\hat{Z}}_{j,\overrightarrow{n}}{\hat{\bar{Z}}}_{j,\overrightarrow{n}}{\hat{\bar{Z}}}_{j,\overrightarrow{n}-\hat{j}}{\hat{Z}}_{j,\overrightarrow{n}-\hat{j}}\right)\\ +\frac{{g}_{{{\rm{4d}}}}^{2}}{{a}^{3}}\sum\limits_{\overrightarrow{n}}{{\rm{Tr}}}\sum\limits_{j < k}\left({\hat{Z}}_{j,\overrightarrow{n}}{\hat{\bar{Z}}}_{j,\overrightarrow{n}}{\hat{Z}}_{k,\overrightarrow{n}}{\hat{\bar{Z}}}_{k,\overrightarrow{n}}+{\hat{Z}}_{j,\overrightarrow{n}}{\hat{\bar{Z}}}_{j,\overrightarrow{n}}{\hat{\bar{Z}}}_{k,\overrightarrow{n}-\hat{k}}{\hat{Z}}_{k,\overrightarrow{n}-\hat{k}}\right.\\ +{\hat{\bar{Z}}}_{j,\overrightarrow{n}-\hat{j}}{\hat{Z}}_{j,\overrightarrow{n}-\hat{j}}{\hat{Z}}_{k,\overrightarrow{n}}{\hat{\bar{Z}}}_{k,\overrightarrow{n}}+{\hat{\bar{Z}}}_{j,\overrightarrow{n}-\hat{j}}{\hat{Z}}_{j,\overrightarrow{n}-\hat{j}}{\hat{\bar{Z}}}_{k,\overrightarrow{n}-\hat{k}}{\hat{Z}}_{k,\overrightarrow{n}-\hat{k}}\\ \left.-2{\hat{Z}}_{j,\overrightarrow{n}}{\hat{Z}}_{k,\overrightarrow{n}+\hat{j}}{\hat{\bar{Z}}}_{j,\overrightarrow{n}+\hat{k}}{\hat{\bar{Z}}}_{k,\overrightarrow{n}}-2{\hat{Z}}_{k,\overrightarrow{n}}{\hat{Z}}_{j,\overrightarrow{n}+\hat{k}}{\hat{\bar{Z}}}_{k,\overrightarrow{n}+\hat{j}}{\hat{\bar{Z}}}_{j,\overrightarrow{n}}\right)\,.\end{array}$$

**Fig. 2:**
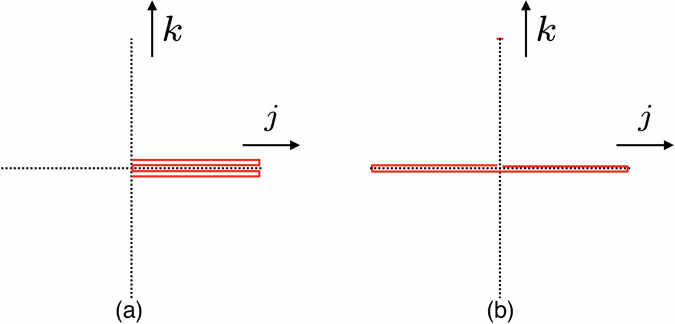
Quartic interaction diagrams. Panel (**a**) shows ${\hat{Z}}_{j,\overrightarrow{n}}{\hat{\bar{Z}}}_{j,\overrightarrow{n}}{\hat{Z}}_{j,\overrightarrow{n}}{\hat{\bar{Z}}}_{j,\overrightarrow{n}}$ and panel (**b**) shows ${\hat{Z}}_{j,\overrightarrow{n}}{\hat{\bar{Z}}}_{j,\overrightarrow{n}}{\hat{\bar{Z}}}_{j,\overrightarrow{n}-\hat{j}}{\hat{Z}}_{j,\overrightarrow{n}-\hat{j}}$. Red solid lines represent links in the lattice.

**Fig. 3:**
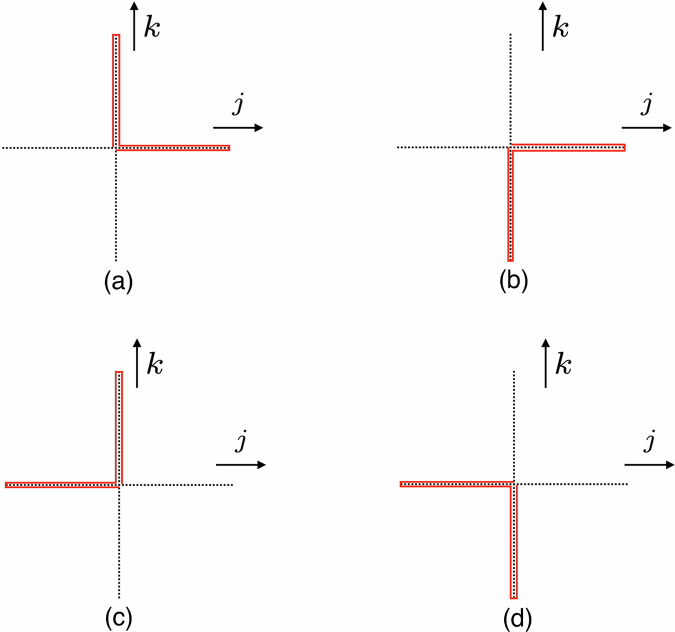
Quartic interactions in the orbifold lattice. Panel (**a**) shows ${\hat{Z}}_{j,\overrightarrow{n}}{\hat{\bar{Z}}}_{j,\overrightarrow{n}}{\hat{Z}}_{k,\overrightarrow{n}}{\hat{\bar{Z}}}_{k,\overrightarrow{n}}$, panel (**b**) shows ${\hat{Z}}_{j,\overrightarrow{n}}{\hat{\bar{Z}}}_{j,\overrightarrow{n}}{\hat{\bar{Z}}}_{k,\overrightarrow{n}-\hat{k}}{\hat{Z}}_{k,\overrightarrow{n}-\hat{k}}$, panel (**c**) shows ${\hat{\bar{Z}}}_{j,\overrightarrow{n}-\hat{j}}{\hat{Z}}_{j,\overrightarrow{n}-\hat{j}}{\hat{Z}}_{k,\overrightarrow{n}}{\hat{\bar{Z}}}_{k,\overrightarrow{n}}$, and panel (**d**) shows ${\hat{\bar{Z}}}_{j,\overrightarrow{n}-\hat{j}}{\hat{Z}}_{j,\overrightarrow{n}-\hat{j}}{\hat{\bar{Z}}}_{k,\overrightarrow{n}-\hat{k}}{\hat{Z}}_{k,\overrightarrow{n}-\hat{k}}$. Red solid lines represent links in the lattice.

In (62), in addition to plaquettes (the final line), there are terms of the forms Figs. 2 and  3. By using the real and imaginary parts of $\hat{Z}$, it is straightforward to rewrite them in the standard form of (1). The number of terms scales as *d*^2^*N*^4^*V*_lattice_.

The additional term $\Delta \hat{H}$ does not change this conclusion. The first term on the right-hand side of (48) is

63 $${\sum }_{j=1}^{3}{{\rm{Tr}}}{\left\vert {\hat{Z}}_{j,\overrightarrow{n}}{\hat{\bar{Z}}}_{j,\overrightarrow{n}}-\frac{a}{2{g}_{{{\rm{4d}}}}^{2}}\right\vert }^{2}={\sum }_{j=1}^{3}{{\rm{Tr}}}\left({\hat{Z}}_{j,\overrightarrow{n}}{\hat{\bar{Z}}}_{j,\overrightarrow{n}}{\hat{Z}}_{j,\overrightarrow{n}}{\hat{\bar{Z}}}_{j,\overrightarrow{n}}-\frac{a}{{g}_{{{\rm{4d}}}}^{2}}{\hat{Z}}_{j,\overrightarrow{n}}{\hat{\bar{Z}}}_{j,\overrightarrow{n}}\right)+{{\rm{const}}}\,.$$

Note that the first term ${\hat{Z}}_{j,\overrightarrow{n}}{\hat{\bar{Z}}}_{j,\overrightarrow{n}}{\hat{Z}}_{j,\overrightarrow{n}}{\hat{\bar{Z}}}_{j,\overrightarrow{n}}$ is in $\hat{H}$ as well. ${{\rm{Tr}}}({\hat{Z}}_{j,\overrightarrow{n}}{\hat{\bar{Z}}}_{j,\overrightarrow{n}})$ has 2*N*^2^ × *d* terms of the form ${\hat{x}}^{2}$. From the second term on the right-hand side of (48), we obtain ${{\rm{Tr}}}({\hat{Z}}_{j,\overrightarrow{n}}{\hat{\bar{Z}}}_{j,\overrightarrow{n}})$ and ${\left[{{\rm{Tr}}}({\hat{Z}}_{j,\overrightarrow{n}}{\hat{\bar{Z}}}_{j,\overrightarrow{n}})\right]}^{2}$. The latter can be written as a sum of 4*N*^4^  × *dV*_lattice_ terms of the form ${\hat{x}}_{1}^{2}{\hat{x}}_{2}^{2}$.

To impose the singlet constraint, we introduce a penalty term associated with the local gauge generators. The generators of gauge transformations at a spatial lattice site $\overrightarrow{n}$ can be written as

64 $${\hat{G}}_{\overrightarrow{n},pq}\equiv {{\rm{i}}}{\sum }_{j=1}^{3}{\left(-{\hat{Z}}_{j,\overrightarrow{n}}{\hat{\bar{P}}}_{j,\overrightarrow{n}}+{\hat{P}}_{j,\overrightarrow{n}}{\hat{\bar{Z}}}_{j,\overrightarrow{n}}-{\hat{\bar{Z}}}_{j,\overrightarrow{n}-\hat{j}}{\hat{P}}_{j,\overrightarrow{n}-\hat{j}}+{\hat{\bar{P}}}_{j,\overrightarrow{n}-\hat{j}}{\hat{Z}}_{j,\overrightarrow{n}-\hat{j}}\right)}_{pq}.$$

As we already mentioned in section “Matrix models”, it is possible in principle to remove SU(*N*) non-singlet states explicitly from the spectrum by adding a penalty term proportional to ${\sum }_{\alpha }{\hat{G}}_{\alpha }^{2}$^44–46^. In this paper, we do not consider this option. If the Hamiltonian time evolution is precise, then it respects SU(*N*) invariance, and hence such a penalty term is not necessary.

### Resource estimate for Suzuki–Trotter time evolution

In this section, we estimate the resources (gate count) needed for Hamiltonian time evolution on a digital quantum computer using the Suzuki–Trotter decomposition. For gauge theories (i.e., matrix model and orbifold lattice) we adopt the extended Hilbert space.

Given the rapid pace of innovation in quantum computing hardware and software, accurately predicting the performance of an orbifold lattice-based code on a quantum computer by, say, 2030, is challenging. Nonetheless, we aim to provide a preliminary upper-bound estimate of its resource requirements to demonstrate how straightforward it is to make such estimates for an orbifold lattice across various lattice geometries, symmetry groups, and matrix models.

As stated above, we do not include a penalty term to enforce the singlet constraint such as ${\hat{G}}_{\alpha }^{2}$ and focus on just one step in the Suzuki–Trotter decomposition. One should keep in mind that often we would need to implement multiple Suzuki–Trotter steps, with their number scaling with the system size and details of interaction terms in order to keep discretization errors under a given threshold^50^. For example, the first-order product formula will need a number of steps scaling quadratically with the total simulation time and inversely with the discretization error we want to achieve^51^. We neglect this complication in the following analysis, and provide resources for a single step.

We point out that a recent work has studied an efficient formulation of Hamiltonian lattice gauge theories in the Kogut–Susskind formalism^52^.

To analyze the structure of our Hamiltonian, it is useful to decompose it into its basic pieces. We can treat the momentum part and the interaction part separately. Because $[{\hat{p}}_{j},{\hat{p}}_{k}]=0$, the momentum part factorizes as

65 $$\exp \left(-{{\rm{i}}}\theta \sum\limits_{j}{\hat{p}}_{j}^{2}\right)={\prod}_{j}\exp \left(-{{\rm{i}}}\theta {\hat{p}}_{j}^{2}\right)\,.$$

Therefore, the total cost is that for one boson times the number of bosons. The interaction part factorizes as well. Schematically, it takes the form

66 $${\prod}_{j,k,l,m}\exp \left(-{{\rm{i}}}\theta {C}_{jklm}\,{\hat{x}}_{j}{\hat{x}}_{k}{\hat{x}}_{l}{\hat{x}}_{m}\right)\,.$$

Here, we ignored the cost of the quadratic and cubic couplings, which are computationally cheaper than the quartic couplings.

We now estimate the computational cost associated with the operator $\exp \left(-{{\rm{i}}}\theta {\hat{x}}_{j}{\hat{x}}_{k}{\hat{x}}_{l}{\hat{x}}_{m}\right)$. As mentioned before, ${\hat{x}}_{j}{\hat{x}}_{k}{\hat{x}}_{l}{\hat{x}}_{m}$ is a sum of tensor products of four Pauli *σ*_*z*_. Schematically, the interaction term is a product of the Pauli rotations,

67 $${\prod}_{pqrs}\exp \left(-{{\rm{i}}}\theta {C}_{pqrs}^{{\prime} }{\hat{\sigma }}_{z,p}{\hat{\sigma }}_{z,q}{\hat{\sigma }}_{z,r}{\hat{\sigma }}_{z,s}\right)\,.$$

Below, we will first demonstrate how each of these Pauli rotations can be implemented using CNOT gates and a single-qubit rotation. For simulations on noisy intermediate-scale quantum (NISQ) devices, it is important to reduce the number of CNOT gates. Next, we will discuss how many T gates are needed to simulate a single-qubit rotation. This is because, for fault-tolerant quantum computing (FTQC), it is important to reduce the number of T gates, which have the largest cost. We begin by examining the counting of CNOT gates. In the following, we demonstrate how these Pauli rotations can be implemented using CNOT gates and a single-qubit rotation. Note that these exponentials are also known as Phase gadgets, or Pauli gadgets^53^, and are well-recognized structures in quantum circuits. They can be manipulated and synthesized efficiently by quantum compilers (e.g., thanks to ZX-calculus^54^). To make this paper self-contained, we will show some useful relations that will help diagonalize these exponentials and decompose them into two-qubit and one-qubit operations. First, we show that

68 $${\hat{\sigma }}_{z,p}{\hat{\sigma }}_{z,q}{\hat{\sigma }}_{z,r}{\hat{\sigma }}_{z,s}={{{\rm{CNOT}}}}_{p,q}{{{\rm{CNOT}}}}_{q,r}{{{\rm{CNOT}}}}_{r,s}{\hat{\sigma }}_{z,s}{{{\rm{CNOT}}}}_{r,s}{{{\rm{CNOT}}}}_{q,r}{{{\rm{CNOT}}}}_{p,q}\,,$$

where CNOT_*p*,*q*_ is the CNOT gate which uses the qubit *p* as the controlled qubit and the qubit *q* as the target qubit:

69 $${{{\rm{CNOT}}}}_{p,q}({\left\vert 0\right\rangle }_{p}{\left\vert 0\right\rangle }_{q})	= {\left\vert 0\right\rangle }_{p}{\left\vert 0\right\rangle }_{q}\,,\qquad {{{\rm{CNOT}}}}_{p,q}({\left\vert 0\right\rangle }_{p}{\left\vert 1\right\rangle }_{q})={\left\vert 0\right\rangle }_{p}{\left\vert 1\right\rangle }_{q}\,,\\ {{{\rm{CNOT}}}}_{p,q}({\left\vert 1\right\rangle }_{p}{\left\vert 0\right\rangle }_{q})	= {\left\vert 1\right\rangle }_{p}{\left\vert 1\right\rangle }_{q}\,,\qquad {{{\rm{CNOT}}}}_{p,q}({\left\vert 1\right\rangle }_{p}{\left\vert 1\right\rangle }_{q})={\left\vert 1\right\rangle }_{p}{\left\vert 0\right\rangle }_{q}\,.$$

Equivalently,

70 $${{{\rm{CNOT}}}}_{p,q}{\left\vert {b}_{p}\right\rangle }_{p}{\left\vert {b}_{q}\right\rangle }_{q}={\left\vert {b}_{p}\right\rangle }_{p}{\left\vert {b}_{p}\oplus {b}_{q}\right\rangle }_{q}\,,$$

where  ⊕ represents exclusive OR, i.e., *b*_*p*_ ⊕ *b*_*q*_ = *b*_*p*_ + *b*_*q*_ mod 2.

Let us see how (68) can be obtained. From (70), it is straightforward to show

71 $$ 	{{{\rm{CNOT}}}}_{r,s} {{{\rm{CNOT}}}}_{q,r}{{{\rm{CNOT}}}}_{p,q}{\left\vert {b}_{p}\right\rangle }_{p}{\left\vert {b}_{q}\right\rangle }_{q}{\left\vert {b}_{r}\right\rangle }_{r}{\left\vert {b}_{s}\right\rangle }_{s} \\ 	 \quad ={\left\vert {b}_{p}\right\rangle }_{p}{\left\vert {b}_{p}\oplus {b}_{q}\right\rangle }_{q}{\left\vert {b}_{p}\oplus {b}_{q}\oplus {b}_{r}\right\rangle }_{r}{\left\vert {b}_{p}\oplus {b}_{q}\oplus {b}_{r}\oplus {b}_{s}\right\rangle }_{s}\,.$$

Since (13) is equivalent to ${\hat{\sigma }}_{z}\left\vert b\right\rangle ={(-1)}^{b}\left\vert b\right\rangle \,,$

72 $$	 {\hat{\sigma }}_{z,s}{{{\rm{CNOT}}}}_{r,s}{{{\rm{CNOT}}}}_{q,r}{{{\rm{CNOT}}}}_{p,q}{\left\vert {b}_{p}\right\rangle }_{p}{\left\vert {b}_{q}\right\rangle }_{q}{\left\vert {b}_{r}\right\rangle }_{r}{\left\vert {b}_{s}\right\rangle }_{s} \\ 	 \quad ={(-1)}^{{b}_{p}\oplus {b}_{q}\oplus {b}_{r}\oplus {b}_{s}}{\left\vert {b}_{p}\right\rangle }_{p}{\left\vert {b}_{p}\oplus {b}_{q}\right\rangle }_{q}{\left\vert {b}_{p}\oplus {b}_{q}\oplus {b}_{r}\right\rangle }_{r}{\left\vert {b}_{p}\oplus {b}_{q}\oplus {b}_{r}\oplus {b}_{s}\right\rangle }_{s}$$

by combining the former and the latter, and by further multiplying with CNOT gates, we obtain

73 $$	 {{{\rm{CNOT}}}}_{p,q}{{{\rm{CNOT}}}}_{q,r}{{{\rm{CNOT}}}}_{r,s}{\hat{\sigma }}_{z,s}{{{\rm{CNOT}}}}_{r,s}{{{\rm{CNOT}}}}_{q,r}{{{\rm{CNOT}}}}_{p,q}{\left\vert {b}_{p}\right\rangle }_{p}{\left\vert {b}_{q}\right\rangle }_{q}{\left\vert {b}_{r}\right\rangle }_{r}{\left\vert {b}_{s}\right\rangle }_{s} \\ 	 \quad ={(-1)}^{{b}_{p}\oplus {b}_{q}\oplus {b}_{r}\oplus {b}_{s}}{\left\vert {b}_{p}\right\rangle }_{p}{\left\vert {b}_{q}\right\rangle }_{q}{\left\vert {b}_{r}\right\rangle }_{r}{\left\vert {b}_{s}\right\rangle }_{s}.$$

On the other hand,

74 $${\hat{\sigma }}_{z,p}{\hat{\sigma }}_{z,q}{\hat{\sigma }}_{z,r}{\hat{\sigma }}_{z,s}{\left\vert {b}_{p}\right\rangle }_{p}{\left\vert {b}_{q}\right\rangle }_{q}{\left\vert {b}_{r}\right\rangle }_{r}{\left\vert {b}_{s}\right\rangle }_{s}={(-1)}^{{b}_{p}\oplus {b}_{q}\oplus {b}_{r}\oplus {b}_{s}}{\left\vert {b}_{p}\right\rangle }_{p}{\left\vert {b}_{q}\right\rangle }_{q}{\left\vert {b}_{r}\right\rangle }_{r}{\left\vert {b}_{s}\right\rangle }_{s}\,.$$

Comparing (73) and (74), we conclude (68).

From (68), we obtain

75 $$	\exp \left(-{{\rm{i}}}\theta {C}_{pqrs}^{{\prime} }{\hat{\sigma }}_{z,p}{\hat{\sigma }}_{z,q}{\hat{\sigma }}_{z,r}{\hat{\sigma }}_{z,s}\right)\\ 	={{{\rm{CNOT}}}}_{p,q}{{{\rm{CNOT}}}}_{q,r}{{{\rm{CNOT}}}}_{r,s}\exp \left(-{{\rm{i}}}\theta {C}_{pqrs}^{{\prime} }{\hat{\sigma }}_{z,s}\right){{{\rm{CNOT}}}}_{r,s}{{{\rm{CNOT}}}}_{q,r}{{{\rm{CNOT}}}}_{p,q}\,,$$

which shows how the Suzuki–Trotter step can be implemented by using CNOT gates and one-qubit rotation gates. See Fig. 4 for the pictorial representation of (75). Note that one can construct rotations with respect to any Pauli operator from (75). For instance, if a Pauli operator contains ${\hat{\sigma }}_{x}$ or ${\hat{\sigma }}_{y}$, one can simply use the change of basis, i.e., $\hat{h}{\hat{\sigma }}_{z}\hat{h}={\hat{\sigma }}_{x}$ and ${\hat{s}}^{{\dagger} }\hat{h}{\hat{\sigma }}_{z}\hat{h}\hat{s}=-{\hat{\sigma }}_{y}$, where $\hat{h}$ is the Hadamard gate and $\hat{s}$ is the phase gate, i.e.,

76 $$\hat{h}=\frac{1}{\sqrt{2}}\left(\begin{array}{cc}1&1\\ 1&-1\end{array}\right)\,,\qquad \hat{s}=\left(\begin{array}{cc}1&0\\ 0&{{\rm{i}}}\end{array}\right)\,.$$

**Fig. 4:**
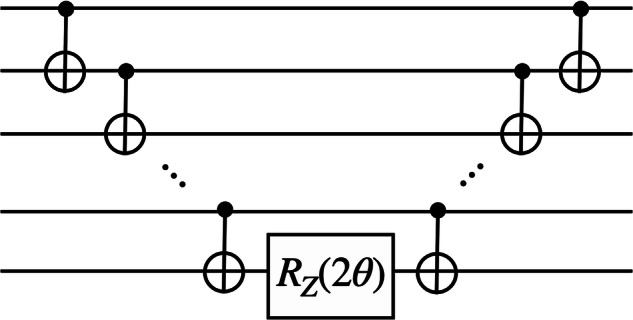
Multi-qubit *Z*-rotation circuit. The unitary $\exp (-{{\rm{i}}}\theta {\hat{\sigma }}_{z}\cdots {\hat{\sigma }}_{z})$ is implemented using controlled-NOT gates and single-qubit *R*_*Z*_(*θ*) rotations, where ${R}_{Z}(\theta )=\exp (-{{\rm{i}}}\theta {\hat{\sigma }}_{z}/2)$.

The number of CNOT gates needed to realize (67) is at most six times the number of combinations *p*, *q*, *r*, *s*, which is 6*Q*^4^ times the number of interaction vertices (~*d*(*d* − 1)*N*^4^ for a matrix model and  ~*d*^2^*N*^4^*V*_lattice_ for an orbifold lattice). Here, *Q* is the number of qubits assigned to each boson. Note that we can reduce the number by taking the products in an appropriate order. For example,

77 $$	 \exp \left(-{{\rm{i}}}\theta {C}_{pqrs}^{{\prime} }{\hat{\sigma }}_{z,p}{\hat{\sigma }}_{z,q}{\hat{\sigma }}_{z,r}{\hat{\sigma }}_{z,s}\right)\cdot \exp \left(-{{\rm{i}}}\theta {C}_{pqr{s}^{{\prime} }}^{{\prime} }{\hat{\sigma }}_{z,p}{\hat{\sigma }}_{z,q}{\hat{\sigma }}_{z,r}{\hat{\sigma }}_{z,{s}^{{\prime} }}\right) \\ 	 = {{{\rm{CNOT}}}}_{p,q}{{{\rm{CNOT}}}}_{q,r}{{{\rm{CNOT}}}}_{r,s}\exp \left(-{{\rm{i}}}\theta {C}_{pqrs}^{{\prime} }{\hat{\sigma }}_{z,s}\right){{{\rm{CNOT}}}}_{r,s}{{{\rm{CNOT}}}}_{q,r}{{{\rm{CNOT}}}}_{p,q} \\ 	 \quad \times {{{\rm{CNOT}}}}_{p,q}{{{\rm{CNOT}}}}_{q,r}{{{\rm{CNOT}}}}_{r,{s}^{{\prime} }}\exp \left(-{{\rm{i}}}\theta {C}_{pqr{s}^{{\prime} }}^{{\prime} }{\hat{\sigma }}_{z,{s}^{{\prime} }}\right){{{\rm{CNOT}}}}_{r,{s}^{{\prime} }}{{{\rm{CNOT}}}}_{q,r}{{{\rm{CNOT}}}}_{p,q} \\ 	 = {{{\rm{CNOT}}}}_{p,q}{{{\rm{CNOT}}}}_{q,r}{{{\rm{CNOT}}}}_{r,s}\exp \left(-{{\rm{i}}}\theta {C}_{pqrs}^{{\prime} }{\hat{\sigma }}_{z,s}\right){{{\rm{CNOT}}}}_{r,s} \\ 	 \quad \times {{{\rm{CNOT}}}}_{r,{s}^{{\prime} }}\exp \left(-{{\rm{i}}}\theta {C}_{pqr{s}^{{\prime} }}^{{\prime} }{\hat{\sigma }}_{z,{s}^{{\prime} }}\right){{{\rm{CNOT}}}}_{r,{s}^{{\prime} }}{{{\rm{CNOT}}}}_{q,r}{{{\rm{CNOT}}}}_{p,q}.$$

In this way, we can eliminate four CNOT gates. This simple relation is very useful. Each $\exp \left(-i\theta {C}_{jklm}{\hat{x}}_{j}{\hat{x}}_{k}{\hat{x}}_{l}{\hat{x}}_{m}\right)$ can be written schematically as

78 $$\exp \left(-{{\rm{i}}}\theta {C}_{jklm}{\hat{x}}_{j}{\hat{x}}_{k}{\hat{x}}_{l}{\hat{x}}_{m}\right)={\prod}_{pqr}\left({\prod}_{s}\exp \left(-{{\rm{i}}}\theta {C}_{pqrs}{\hat{\sigma }}_{z,p}{\hat{\sigma }}_{z,q}{\hat{\sigma }}_{z,r}{\hat{\sigma }}_{z,s}\right)\right)$$

and CNOT_*p*,*q*_s and CNOT_*q*,*r*_s in ${\prod }_{s}\exp \left(-{{\rm{i}}}\theta {C}_{pqrs}{\hat{\sigma }}_{z,p}{\hat{\sigma }}_{z,q}{\hat{\sigma }}_{z,r}{\hat{\sigma }}_{z,s}\right)$ cancel out, leaving only two CNOT_*p*,*q*_s and two CNOT_*q*,*r*_s; for each set of (*p*, *q*, *r*), the number of CNOT gates left in $\left({\prod }_{s}\cdots \,\right)$ is 2*Q* + 4 rather than 6*Q*.

The combination of CNOT gates and single-qubit rotation gates forms a universal gate set, enabling the construction of any quantum algorithm using only these gates. This gate set is especially important in the NISQ era, where quantum computations are performed on physical qubits without the benefit of error correction. In NISQ devices, all gates are implemented directly on the physical qubits. Note that there exist different physical implementations of qubit gates and some platforms, like the H-series hardware by Quantinuum^55^, can implement directly arbitrary angle two-qubit gates ${e}^{-{{\rm{i}}}{\theta }_{pq}{\hat{\sigma }}_{z,p}{\hat{\sigma }}_{z,q}}$ using a single laser pulse.

Without error corrections, the fidelity of quantum gates becomes a critical factor in determining how well a computation can be performed. Notably, the fidelity of two-qubit gates, like the CNOT gate, is typically much lower than that of single-qubit gates. As a result, errors accumulate more rapidly when a quantum circuit relies heavily on two-qubit gates, which can significantly degrade the overall performance of the algorithm.

Given the lower fidelity associated with two-qubit gates, it is reasonable to consider the number of CNOT gates as a key computational resource when assessing the efficiency and accuracy of quantum simulations in the NISQ regime. The CNOT gate count serves as a useful metric for estimating how error-prone a quantum algorithm might be on current hardware. By minimizing the number of CNOT gates in a circuit, we can reduce the potential for error accumulation, thereby improving the fidelity of the computation.

We now turn to the counting of T gates. In FTQC, the computational paradigm shifts significantly compared to NISQ systems. In FTQC, error correction is employed through the use of logical qubits, which are constructed from multiple physical qubits. This allows for the protection of quantum information from noise and errors, enabling more robust and scalable quantum computations. As a result, the focus on computational cost changes: the number of CNOT gates, which plays a critical role in NISQ systems, is no longer the primary factor determining the computational expense.

The most commonly used universal gate set in FTQC is the combination of Clifford gates and the T gates. The T gate is defined by

$$\hat{T}=\left(\begin{array}{cc}1&0\\ 0&\exp \left(\frac{{{\rm{i}}}\pi }{4}\right)\end{array}\right)\,.$$

Clifford gates, including the Hadamard, phase, and CNOT gates, are generally not resource-intensive in various error-correcting codes, such as the surface code^56^. They can often be implemented efficiently through techniques like code deformation and lattice surgery^57,58^. Additionally, Clifford gates can be pushed to the end of a quantum circuit, where they can be seamlessly absorbed into Pauli measurements^59^, further optimizing resource usage.

In contrast, non-Clifford gates, such as the T gate, are typically much more resource-intensive. A fault-tolerant implementation of the T gate often involves gate teleportation, which relies on specialized resource states known as magic states^60,61^. However, producing high-fidelity magic states is considerably costly. As a result, the T gate count has become a standard metric for estimating resource requirements in FTQC. There has been significant progress in improving the efficiency of magic state distillation; for instance, see the recent advancements in ref. ^62^.

In order to switch from the universal gate set {CNOT, single-qubit rotations} to {Clifford, T}, each rotation gate—specifically the *R*_*Z*_ gate, ${R}_{Z}(\theta )=\exp \left(-\frac{{{\rm{i}}}\theta }{2}{\hat{\sigma }}_{z}\right)$—needs to be approximated using a combination of T gates and single-qubit Clifford gates. For instance, ref. ^63^ demonstrated that each *R*_*Z*_ rotation can typically be approximated with a T gate count of $3\log \left(1/\epsilon \right)+O\,\left(\log \log \left(1/\epsilon \right)\right)$, where *ϵ* represents the desired accuracy of the approximation. We also point out that an intermediate framework between NISQ and FTQC can be implemented, which is partially fault-tolerant^64^. This framework can be used to compile the type of Trotter circuits we are considering in this section^65^. The *R*_*Z*_ gate can be written in terms of more elementary one-qubit gates, among which the T gate is usually the most costly one. The typical T gate count per *R*_*Z*_ gate will fall within the range of 10–50^66^, depending on factors such as the rotation angle, desired accuracy, and the specific algorithm used to decompose the *R*_*Z*_ gate into T gates and Clifford gates. In the following, we denote the T gate count per *R*_*Z*_ as *T*_typ_ and use *T*_typ_ = 10–50^66,67^.

We now estimate the computational cost associated with the operator $\exp \left(-{{\rm{i}}}\theta {\hat{p}}^{2}\right)$. One of the advantages of the orbifold lattice over the Kogut–Susskind formulation is that the Fourier transform is straightforward. By switching from the coordinate basis to the momentum basis via Fourier transform, we can diagonalize the kinetic terms. Earlier in this paper, we showed two options, (20) and (21). The second option (21) is obtained by omitting the higher-order terms of the Taylor expansion of (20). Here, we choose the second option, because these two options give the same results when the truncation is removed, but higher powers of Pauli *σ*_*z*_ appear, and more gates are needed for the first option. Then, $\hat{p}$ in the momentum basis takes essentially the same form as $\hat{x}$ in the coordinate basis. Specifically, $\hat{p}$ is a linear sum of *Q* Pauli *σ*_*z*_ (we call them ${\hat{\sigma }}_{z,1},\cdots \,,{\hat{\sigma }}_{z,Q}$), and therefore ${\hat{p}}^{2}$ is a combination of ${\hat{\sigma }}_{z,j}{\hat{\sigma }}_{z,k}$ (1≤*j* < *k*≤*Q*). We can write ${e}^{-{{\rm{i}}}\theta {\hat{p}}^{2}}$ as

79 $$\exp \left(-{{\rm{i}}}\theta {\hat{p}}^{2}\right)={\prod}_{j < k}\exp \left(-{{\rm{i}}}\theta {C}_{jk}{\hat{\sigma }}_{z,j}{\hat{\sigma }}_{z,k}\right)\,.$$

Each term in the product can be written in terms of CNOT gates and one-qubit gates as before:

80 $$\exp \left(-{{\rm{i}}}\theta {C}_{jk}{\hat{\sigma }}_{z,j}{\hat{\sigma }}_{z,k}\right)={{{\rm{CNOT}}}}_{j,k}\exp \left(-{{\rm{i}}}\theta {C}_{jk}{\hat{\sigma }}_{z,k}\right){{{\rm{CNOT}}}}_{j,k}\,.$$

In this case, there is no cancellation between CNOT gates. $$2\times \left(\left(\begin{array}{c}Q\\ 2\end{array}\right)\right)=Q(Q-1)$$ CNOT gates are needed for each boson. The number of *R*_*Z*_ rotations is *Q*(*Q* − 1)/2, and the number of T gates in FTQC is $O\left(Q(Q-1)\right)$.

**Fig. 5:**
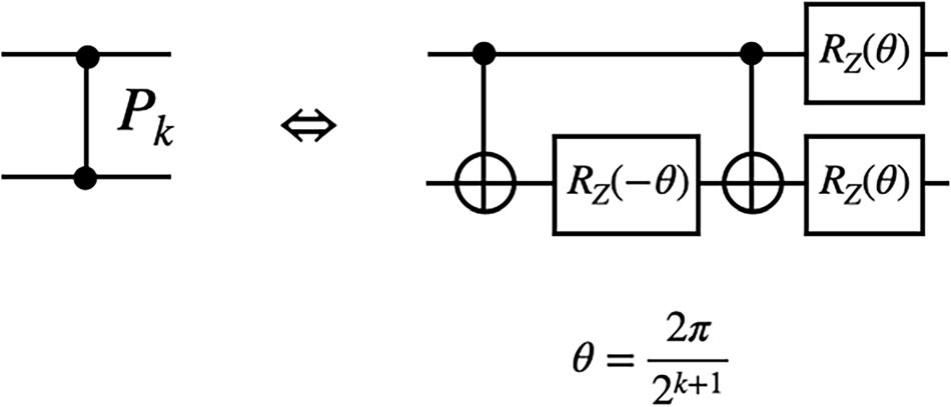
Quantum Fourier transform circuit. The circuit implements the discrete Fourier transform on qubit basis states using controlled-phase gates and Hadamard gates.

The cost of implementing the diagonal kinetic terms (79) is dominated by the need to perform quantum Fourier transforms between the potential terms and the kinetic terms. In Fig. 5, we show the exact quantum Fourier transform circuit.

The quantum Fourier transform circuit consists of Hadamard gates and controlled-phase gates. A controlled-phase gate is defined as

81 $${P}_{k}=\left(\begin{array}{cccc}1&0&0&0\\ 0&1&0&0\\ 0&0&1&0\\ 0&0&0&\exp \left(\frac{2\pi {{\rm{i}}}}{{2}^{k}}\right)\\ \end{array}\right)\,.$$

**Fig. 6:**
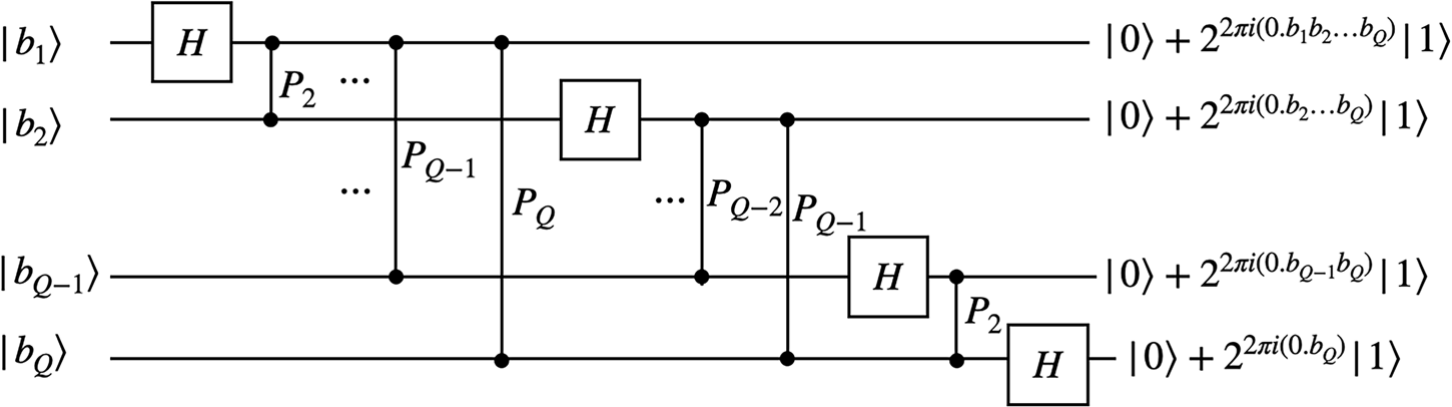
Controlled-phase gate decomposition. The decomposition uses CNOT gates and single-qubit rotation gates (equivalent up to a global phase) to illustrate how controlled-phase shifts can be implemented within a universal gate set.

Up to a global phase, a controlled-phase gate is decomposed into CNOT gates and 1 qubit rotation gates *R*_*Z*_(*θ*) as in Fig. 6.

The quantum Fourier transform circuit (Fig. 5) has *Q*(*Q* − 1)/2 controlled-phase gates. In terms of CNOT gates, the two-qubit gate count is *Q*(*Q* − 1), and the number of *R*_*Z*_ rotation gates is 3*Q*(*Q* − 1)/2. An approximate quantum Fourier transform can also be used by truncating rotations with angles below a specified threshold. With this approach, both the T gate and CNOT gate counts can be reduced to $O(Q\log Q)$^68^.

In the NISQ era, it may be advantageous to avoid using the Fourier transform altogether and instead operate solely in the coordinate basis. For the coordinate basis, $\hat{{{\mathcal{S}}}}\equiv {\sum }_{n}\left(\left\vert n+1\right\rangle \left\langle n\right\vert +\left\vert n\right\rangle \left\langle n+1\right\vert \right)$ can be written as

82 $$\hat{{{\mathcal{S}}}}={\hat{\sigma }}_{x}\,$$

for *Q* = 1,

83 $$\hat{{{\mathcal{S}}}}={\hat{\sigma }}_{x}\otimes {{{\bf{1}}}}_{2}+\frac{{\hat{\sigma }}_{x}\otimes {\hat{\sigma }}_{x}+{\hat{\sigma }}_{y}\otimes {\hat{\sigma }}_{y}}{2}\,.$$

for *Q* = 2,

84 $$\hat{{{\mathcal{S}}}} = 	 {\hat{\sigma }}_{x}\otimes {{{\bf{1}}}}_{2}\otimes {{{\bf{1}}}}_{2}+\frac{{\hat{\sigma }}_{x}\otimes {\hat{\sigma }}_{x}\otimes {{{\bf{1}}}}_{2}+{\hat{\sigma }}_{y}\otimes {\hat{\sigma }}_{y}\otimes {{{\bf{1}}}}_{2}}{2} \\ 	 + \frac{{\hat{\sigma }}_{x}\otimes {\hat{\sigma }}_{x}\otimes {\hat{\sigma }}_{x}-{\hat{\sigma }}_{x}\otimes {\hat{\sigma }}_{y}\otimes {\hat{\sigma }}_{y}+{\hat{\sigma }}_{y}\otimes {\hat{\sigma }}_{x}\otimes {\hat{\sigma }}_{y}+{\hat{\sigma }}_{y}\otimes {\hat{\sigma }}_{y}\otimes {\hat{\sigma }}_{x}}{4}$$

for *Q* = 3,

85 $$\hat{{{\mathcal{S}}}} = 	 {\hat{\sigma }}_{x}\otimes {{{\bf{1}}}}_{2}\otimes {{{\bf{1}}}}_{2}\otimes {{{\bf{1}}}}_{2}+\frac{{\hat{\sigma }}_{x}\otimes {\hat{\sigma }}_{x}\otimes {{{\bf{1}}}}_{2}\otimes {{{\bf{1}}}}_{2}+{\hat{\sigma }}_{y}\otimes {\hat{\sigma }}_{y}\otimes {{{\bf{1}}}}_{2}\otimes {{{\bf{1}}}}_{2}}{2}\\ 	 + \frac{{\hat{\sigma }}_{x}\otimes {\hat{\sigma }}_{x}\otimes {\hat{\sigma }}_{x}\otimes {{{\bf{1}}}}_{2}-{\hat{\sigma }}_{x}\otimes {\hat{\sigma }}_{y}\otimes {\hat{\sigma }}_{y}\otimes {{{\bf{1}}}}_{2}+{\hat{\sigma }}_{y}\otimes {\hat{\sigma }}_{x}\otimes {\hat{\sigma }}_{y}\otimes {{{\bf{1}}}}_{2}+{\hat{\sigma }}_{y}\otimes {\hat{\sigma }}_{y}\otimes {\hat{\sigma }}_{x}\otimes {{{\bf{1}}}}_{2}}{4}\\ 	 + \frac{1}{8}\left\{{\hat{\sigma }}_{x}\otimes {\hat{\sigma }}_{x}\otimes {\hat{\sigma }}_{x}\otimes {\hat{\sigma }}_{x}+{\hat{\sigma }}_{x}\otimes {\hat{\sigma }}_{x}\otimes {\hat{\sigma }}_{y}\otimes {\hat{\sigma }}_{y}+{\hat{\sigma }}_{x}\otimes {\hat{\sigma }}_{y}\otimes {\hat{\sigma }}_{x}\otimes {\hat{\sigma }}_{y}-{\hat{\sigma }}_{x}\otimes {\hat{\sigma }}_{y}\otimes {\hat{\sigma }}_{y}\otimes {\hat{\sigma }}_{x}\right.\\ 	 + \left.{\hat{\sigma }}_{y}\otimes {\hat{\sigma }}_{x}\otimes {\hat{\sigma }}_{x}\otimes {\hat{\sigma }}_{y}-{\hat{\sigma }}_{y}\otimes {\hat{\sigma }}_{x}\otimes {\hat{\sigma }}_{y}\otimes {\hat{\sigma }}_{x}-{\hat{\sigma }}_{y}\otimes {\hat{\sigma }}_{y}\otimes {\hat{\sigma }}_{x}\otimes {\hat{\sigma }}_{x}-{\hat{\sigma }}_{y}\otimes {\hat{\sigma }}_{y}\otimes {\hat{\sigma }}_{y}\otimes {\hat{\sigma }}_{y}\right\}$$

for *Q* = 4, and so on. In general, we can write $\hat{{{\mathcal{S}}}}$ as a sum of 2^*q*−1^ Pauli strings of length *q* consisting of ${\hat{\sigma }}_{x}$ and ${\hat{\sigma }}_{y}$, where *q* runs from 1 to *Q*. This decomposition can be practical when *Q* is relatively small.

A potential advantage of the implementation in the coordinate basis is that the truncation effect can be quantitatively estimated by using a classical sampling method^39^. We now combine all the preceding components to estimate the computational resources required for Hamiltonian time evolution. Specifically, we evaluate the cost of one Suzuki–Trotter step for the scalar QFT, matrix model, and Yang–Mills theory. We focus on the asymptotic behavior with respect to the system size parameters, i.e., matrix size *N*, lattice volume *V*_lattice_ = *L*^*d*^, and truncation parameter *Q*. Again, we neglect the cost related to reaching a constant accuracy.

We begin with the case of scalar quantum field theory. The number of bosons is *V*_lattice_ with one boson on each lattice site. By assigning *Q* qubits to each boson, a total of *V*_lattice_*Q* qubits are utilized. Recently, ref. ^69^ has studied this case with *Q* = 2 and *V*_lattice_ = *L* = 60 in (1 + 1) dimensions, for a total of 120 qubits on a near-term quantum device.

The potential term of the Hamiltonian (23) consists of quadratic and quartic terms. The former consists of two-*σ*_*z*_ couplings, while the latter consists of two-*σ*_*z*_ couplings and four-*σ*_*z*_ couplings.

Let us focus on the four-*σ*_*z*_ couplings. The number of such couplings is $$\left(\begin{array}{c}Q\\ 4\end{array}\right)\times {V}_{{{\rm{lattice}}}} \sim {Q}^{4}{V}_{{{\rm{lattice}}}}$$. As explained above, Suzuki–Trotter time evolution with respect to each of these couplings can be written by using six CNOT gates and one *R*_*Z*_ rotation acting on one of the four qubits. By taking appropriate ordering, about 2/3 of CNOT gates cancel (see a comment right after (78)). The *R*_*Z*_ gate can be built using more elementary one-qubit gates, including *T*_typ_ = 10–50 T gates. Therefore, we are left with *O*(*Q*^4^*V*_lattice_) CNOT gates, *O*(*T*_typ_*Q*^4^*V*_lattice_) T gates, and *O*(*Q*^4^*V*_lattice_) one-qubit gates simpler than the T gate. Next, we consider the kinetic terms. If we use an approximate quantum Fourier transform to change to the momentum basis, the cost in terms of both CNOT gates and T gates is $O({V}_{{{\rm{lattice}}}}Q\log Q)$. As we saw, *Q*(*Q* − 1) CNOT gates and about *T*_typ_*Q*(*Q* − 1)/2 T gates are needed in the momentum basis for each boson. Multiplying by the number of bosons, we obtain the total cost as *O*(*Q*^2^*V*_lattice_) CNOT gates, *O*(*T*_typ_*Q*^2^*V*_lattice_) T gates, and *O*(*Q*^2^*V*_lattice_) one-qubit gates, simpler than the T gate.

Next, we consider the case of the matrix model. The number of bosons scales as *dN*^2^. Typically, we are interested in the large-*N* limit, where the difference between *N*^2^ and *N*^2^ − 1 is not important. We assign *Q* qubits to each boson, and hence *dN*^2^*Q* qubits are used in total. Firstly, we consider the interaction terms. As we saw in Sec. Matrix Models, the number of quartic interactions of the form ${\hat{x}}_{j}{\hat{x}}_{k}{\hat{x}}_{l}{\hat{x}}_{m}$ increases as *d*(*d* − 1)*N*^4^. Each $\hat{x}$ can be expressed by using *Q* Pauli ${\hat{\sigma }}_{z}$’s, and hence, there are  ~ *d*(*d* − 1)*N*^4^*Q*^4^ quartic couplings of ${\hat{\sigma }}_{z}$’s.

Furthermore, Suzuki–Trotter time evolution with respect to each of these four-*σ*_*z*_ couplings can be written by using six CNOT gates and about one-qubit gate (or *T*_typ_ T gates). We need  ~ *d*(*d* − 1)*N*^4^*Q*^4^ CNOT gates and  ~ *T*_typ_ × *d*(*d* − 1)*N*^4^*Q*^4^ T gates.

Because the number of both one- and two-qubit gates scales as *d*(*d* − 1)*N*^4^*Q*^4^, we can estimate the depth of the circuit by simply dividing this number by the number of qubits *dN*^2^*Q*, which leads to the conclusion that the depth scales like (*d* − 1)*N*^2^*Q*^3^.

As for the kinetic terms, we consider the implementation in the momentum basis with the quantum Fourier transform. As we saw, *Q*(*Q* − 1) CNOT gates and *T*_typ_*Q*(*Q* − 1) T gates are needed for each boson, once we are in the momentum basis. Multiplying by the number of bosons, we obtain the cost as *dN*^2^*Q*(*Q* − 1) CNOT gates and *T*_typ_*dN*^2^*Q*(*Q* − 1) T gates. In addition, we add the cost of the quantum Fourier transform as we did before: if we use an approximate quantum Fourier transform, this cost is $O(d{N}^{2}Q\log Q)$ CNOT (and T) gates.

In total, the cost for the kinetic term is negligible compared to the cost for the interaction terms.

Finally, we consider the case of the orbifold lattice. The number of bosons and logical qubits used for encoding them are 2*N*^2^*dV*_lattice_ and 2*N*^2^*dV*_lattice_*Q*, respectively. To see the cost for one Suzuki–Trotter step of the interaction part, we combine the results in this section and Sec. Orbifold Lattice. There are  ~ *d*^2^*V*_lattice_*N*^4^ terms quartic in $\hat{x}$. We can write them using ${\hat{\sigma }}_{z}$, leading to  ~ *Q*^4^ CNOT gates and  ~ *T*_typ_ × *Q*^4^ T gates for each quartic interaction. In total, we need  ~ *d*^2^*V*_lattice_*N*^4^*Q*^4^ CNOT gates and  ~ *T*_typ_ × *d*^2^*V*_lattice_*N*^4^*Q*^4^ T gates.

As for the kinetic terms, we consider the implementation in the momentum basis with the quantum Fourier transform. Then, we need *N*^2^*dV*_lattice_*Q*(*Q* − 1) CNOT gates and  ~ *T*_typ_ × *N*^2^*dV*_lattice_*Q*(*Q* − 1) T gates once we are in the momentum basis. The cost of the quantum Fourier transform itself is $O({N}^{2}d{V}_{{{\rm{lattice}}}}Q\log Q)$ CNOT (and T) gates.

In total, the cost for the kinetic term is negligible compared to the cost for the interaction terms.

## Discussion

In this paper, we provided a universal framework for the quantum simulation of SU(*N*) Yang–Mills theories with arbitrary *N*, arbitrary spatial dimensions, and arbitrary lattice sizes, adopting the orbifold lattice formulation, taking the Hamiltonian time evolution as an example. Technical difficulties associated with the Kogut–Susskind Hamiltonian, which researchers struggled for years to solve on a case-by-case basis—such as the definition of the coordinate basis (magnetic basis), the quantum Fourier transform, and the realization of complicated interactions in the momentum basis (electric basis) expressed by the Clebsh–Gordan coefficient in terms of quantum gates—simply do not exist in the orbifold lattice formulation. This is a consequence of a simple and universal form (1) that is common among many theories. In this paper, we did not consider theories with fermions; see ref. ^37^ for the orbifold lattice construction of QCD, i.e., Yang–Mills theory with fermions in the fundamental representation. We have focused here on bosonic theories without fermions, but the inclusion of fermions is straightforward, and we plan to discuss quantum simulation with fermions in a separate paper in the near future.

We know explicitly how the orbifold Hamiltonian can be programmed on a quantum computer for any *N*, any dimensions, and any lattice size. Furthermore, we need only standard, well-established tools in the field of quantum computing. As a warm-up example, we also considered the Yang–Mills matrix model and the more standard scalar quantum field theory on a lattice. It was straightforward to write a circuit for the unitary Hamiltonian evolution operator explicitly in terms of CNOT gates and one-qubit gates and count the number of gates.

We used the extended Hilbert space ${{{\mathcal{H}}}}_{{{\rm{ext}}}}$ that contains SU(*N*) non-singlets. This is a standard approach, and we believe one should not stick to the projection to singlet Hilbert space ${{{\mathcal{H}}}}_{{{\rm{inv}}}}$ because both ${{{\mathcal{H}}}}_{{{\rm{ext}}}}$ and ${{{\mathcal{H}}}}_{{{\rm{inv}}}}$ lead to mathematically equivalent formulations and, on ${{{\mathcal{H}}}}_{{{\rm{inv}}}}$, one cannot even define the coordinate and momentum operators. It is often claimed that the use of ${{{\mathcal{H}}}}_{{{\rm{ext}}}}$ is too costly because the dimension is exponentially larger. Such a claim might be missing an important point: although it is true that brute-force computations on a classical computer are hard if the dimension of the Hilbert space is exponentially larger, on a quantum computer, it only requires a moderate number of additional qubits. Adding longitudinal modes just increases the number of qubits by 50% in three spatial dimensions. However, with this overhead, the structure of the Hilbert space and quantum circuits simplify drastically, outweighing the increase in *space* resources. Because our target is a quantum simulation and not a classical simulation, it is therefore advantageous to use the extended Hilbert space. Note also that, if one wants to remove non-singlet modes explicitly from the spectrum, one can add a penalty term such as ${\sum }_{\overrightarrow{n}}{{\rm{Tr}}}{\hat{G}}_{\overrightarrow{n}}^{2}$ to the Hamiltonian^44–46^. We also note that it is rather straightforward to construct many singlet states in the extended Hilbert space because the confined vacuum is a singlet and any state that is obtained by acting with singlet operators on it is also a singlet. Many singlet operators can be constructed by using Wilson loops, which are traces of products of link variables ${\hat{Z}}_{j,\overrightarrow{n}}$, ${\hat{\bar{Z}}}_{j,\overrightarrow{n}}$ along a closed contour. Regarding the Hamiltonian time evolution, for example, the only thing we need to keep the states in the singlet sector is the precision of the unitary time evolution, which is made significantly more tractable by the simplicity of the simulation scheme in the orbifold lattice formulation.

In this context, it is important to note that the violation of gauge symmetry is suppressed exponentially with regard to the truncation level *Λ*; see Supplementary Note 2. Furthermore, note that the violation of gauge symmetry is only one of many possible truncation effects. Our universal approach leads to efficient suppression of all kinds of truncation effects, because we can more easily remove the truncation. On the other hand, imposing exact gauge symmetry at truncated level makes the Hamiltonian complicated and makes it harder to remove truncation. Also, as a trade-off, other kinds of truncation effects can become severer^70^. Finding a good gauge-invariant basis that circumvents such an unwanted trade-off would be as challenging as finding the energy eigenstates.

We also note that the key role of gauge symmetry is to introduce massless vector fields compatible with Lorentz symmetry without having negative-norm states. In lattice Hamiltonian formulations, even when the truncation in the Hilbert space is removed, Lorentz symmetry is broken at finite lattice spacing. Adherence to the single Hilbert space can make it difficult to go to sufficiently small lattice spacing and hence can lead to larger breaking of Lorentz symmetry, spoiling the original motivation of gauge symmetry.

We note that the scalar *ϕ*^4^ theory considered in ref. ^43^ has the same simple form (1). (We show this in the section “Scalar quantum field theory”) The only difference is in the detail of the polynomial $V(\hat{x})$. In this sense, the implementation of the Hamiltonian of SU(*N*) Yang–Mills theory on a quantum computer is not more complicated than that of scalar *ϕ*^4^ theory. We can see the essence already in much simpler models, e.g., the anharmonic oscillator.

In Table 1, we show a summary of qubit and T gate requirements for the Hamiltonian time evolution of the theories we study in this paper. Here, *V*_lattice_ is the lattice volume (number of lattice sites), *d* is the spatial dimension (for scalar QFT and orbifold YM) or the number of matrices (for matrix model), *N* characterizes the gauge group SU(*N*), and *Q* is the number of qubits assigned to each bosonic degree of freedom. For fault-tolerant quantum simulations, ‘the number of qubits’ means the number of logical qubits. As for the gate counts, we showed only the number for one time step in the Suzuki–Trotter decomposition, and we showed only scaling with the parameters characterizing the system size. More details are explained in the following sections. Note that the number of gates in this table is proportional to the number of interaction terms in the Hamiltonian. Each interaction term can be implemented efficiently so that the simulation cost does not become large.

**Table 1:** Resources for a Suzuki–Trotter step

	Number of qubits	# T gates in $V(\hat{x})$ term	# T gates in ${\hat{p}}^{2}$ term
Scalar QFT	*V*_lattice_*Q*	$${V}_{{{\rm{lattice}}}}\left(\begin{array}{c}Q\\ 4\end{array}\right)$$	*V*_lattice_*Q*(*Q* − 1)
Matrix Model	*dN*^2^*Q*	*d*(*d* − 1)*N*^4^*Q*^4^	*dN*^2^*Q*(*Q* − 1)
Orbifold YM	2*dN*^2^*V*_lattice_*Q*	*d*^2^*V*_lattice_*N*^4^*Q*^4^	*N*^2^*dV*_lattice_*Q*(*Q* − 1)

The table summarizes qubit counts and T gate counts for Hamiltonian time evolution of the considered theories, which share the universal Hamiltonian form. Detailed derivations are provided in the section “Resource estimate for Suzuki–Trotter time evolution”.

Given the striking simplicity of the Hamiltonians, it is important to investigate efficient simulation techniques for orbifold lattice theory and matrix model systematically. Note that even the Hamiltonian of the scalar *ϕ*^4^ theory belongs to the same class (specifically, it takes the same simple form (1)) and hence a very large class of theories can be studied in a unified manner. It is also interesting to try simulations on real quantum devices. A good starting point is randomly-coupled spin systems^71–76^. Among them, the spin-XY4 model^74^ consists of four-body random coupling of ${\hat{\sigma }}_{x}$ and ${\hat{\sigma }}_{y}$. We can replace ${\hat{\sigma }}_{y}$ with ${\hat{\sigma }}_{z}$ without any change to the physical properties, and ${\hat{\sigma }}_{x}$ can be written by using ${\hat{\sigma }}_{z}$ and the Hadamard gate $\hat{h}$ according to $\hat{h}{\hat{\sigma }}_{z}\hat{h}={\hat{\sigma }}_{x}$. Therefore, quantum simulation of the spin-XY4 model can be a good exercise toward the simulation of matrix models and gauge theories on an orbifold lattice. A simplified version of the matrix model studied in ref. ^77^ or the anharmonic oscillator (3) (see ref. ^40^ for the analysis of this model in the context of quantum simulation) would also be a good target for the first quantum simulation on real devices.

If we use four logical qubits for a single anharmonic oscillator (one boson), for example, then the coordinate operator is

86 $$\hat{x}=-{\delta }_{x}\cdot \left(\frac{{\hat{\sigma }}_{z;1}}{2}+2\cdot \frac{{\hat{\sigma }}_{z;2}}{2}+4\cdot \frac{{\hat{\sigma }}_{z;3}}{2}+8\cdot \frac{{\hat{\sigma }}_{z;4}}{2}\right)\,,$$

and ${\hat{x}}^{4}$ contains ${\hat{\sigma }}_{z;1}\otimes {\hat{\sigma }}_{z;2}\otimes {\hat{\sigma }}_{z;3}\otimes {\hat{\sigma }}_{z;4}$. This is exactly the interaction we need to describe a wide class of theories, including Yang–Mills theory, as we have argued in this paper. To study a larger system with more bosons, we need to add more logical qubits. However, we only have to add the same four-qubit gates acting on different sets of qubits. Furthermore, to switch to the momentum basis, we only have to perform the same quantum Fourier transform to different sets of qubits that describe different bosons. In the literal sense, we know how to scale up such a simulation systematically when more logical qubits are available. Therefore, it would be already meaningful if we could use a few logical qubits and demonstrate the precise Hamiltonian time evolution, even if the system size is small and there is no quantum advantage. In the context of NISQ, we have recently witnessed how the simple protocol we propose can be enhanced by variational circuits and used to study particle scattering in a (1 + 1) dimensional Scalar Field Theory with up to 120 qubits^69^. This is a testament to the fact that there are no theoretical obstacles in going to more complicated theories and to higher dimensions using our universal framework.

It is also important to identify the types of hardware suitable for quantum simulations. The large-*N* limit of the matrix model involves nonlocal interactions between matrix entries, and hence, the truncated theory has nonlocal interactions between qubits. Trapped-ion quantum computers would be suitable for such a Hamiltonian because any pair of qubits ( = ions) can be brought close to each other, and nonlocal interaction can naturally be realized. On the other hand, the orbifold lattice Hamiltonian has a local structure associated with the spatial lattice, although there is some non-locality that increases with the number of colors *N* and truncation level *Λ* = 2^*Q*^, and hence, quantum hardware with qubits at fixed locations, such as the superconducting qubit machines, may also perform well. We also note that standard lattice simulations on classical devices are a powerful tool for studying the non-perturbative features of the orbifold-lattice Hamiltonian, because the Euclidean counterpart can be obtained by employing a spacetime lattice and taking the continuum limit along the time direction. Such a method was applied for the Kogut–Susskind Hamiltonian, by using Wilson’s action on an anisotropic lattice. Just as an incomplete list: refs. ^78,79^ related Hamiltonian time evolution with finite Suzuki–Trotter step to simulations on Euclidean anisotropic lattices. Their main interest was in the discretization effects. If the Suzuki–Trotter step is sent to zero, then their setup is the same as ours. Ref. ^80^ studied the renormalization of the anisotropy ratio in (2 + 1)-dimensional QED on an anisotropic lattice. Such a renormalization is directly related to the tuning of the coupling as a function of spatial lattice spacing, which is needed for the restoration of Lorentz symmetry. See also ref. ^81^.

In this paper, we focused on digital simulations with qubits. However, the simplifications coming from the use of non-compact variables are not limited to this particular setup. For example, it is straightforward to write the truncated Hamiltonian in terms of qudits. Furthermore, quantum simulation with continuous variables (see ref. ^82^ for a review and refs. ^83,84^ for a recent application to quantum field theory) is potentially a good framework to simulate orbifold lattices and matrix models. Another potentially promising route is to engineer an analog simulator to be described by the same Hamiltonian. It would be an interesting research avenue to identify the right setup that allows quantum simulations before the arrival of fault-tolerant quantum computers.

As an important side comment, we note that the simplicity of the orbifold lattice is connected to the emergent geometry. The orbifold lattice emerges from a matrix model with a certain background, via dimensional deconstruction^47,49^. A related example is the emergence of D2-branes from D0-branes via the Myers effect^85^ that enables us to describe a (2 + 1)-dimensional theory by using a (0 + 1)-dimensional theory, whose prototype dates back to the non-commutative torus in the twisted Eguchi–Kawai model^86^. In these examples, theories on emergent spaces inherit simple structures from the original theories^87^. We could say that nature is smarter than humans, and hence dynamically-generated spatial dimensions can have better properties than a lattice crafted by humans. Holographic dualities provide us with even more profound examples: gravitational geometries emerge from non-gravitational theories, providing us with simple Hamiltonians of the non-gravitational theories. We contend that the quantum simulation of quantum field theory should be considered within the broader context of the web of dualities and emergent geometry.

To conclude this paper, we would like to refer to a famous essay on AI research *The Bitter Lesson* written by Rich Sutton in 2019. The opening phrase of this essay is “*The biggest lesson that can be read from 70 years of AI research is that general methods that leverage computation are ultimately the most effective, and by a large margin.”* Then, it continues as *"[...] Seeking an improvement that makes a difference in the shorter term, researchers seek to leverage their human knowledge of the domain, but the only thing that matters, in the long run, is the leveraging of computation. [...] And the human-knowledge approach tends to complicate methods in ways that make them less suited to taking advantage of general methods leveraging computation. [...] ”* The same lesson applied to classical computing for quantum field theory. Indeed, revolutionary algorithms such as Metropolis^88^ and Hybrid Monte Carlo^89^ do not assume too many details of the theories, and the same methods can be used for a wide class of theories. Similar lessons may apply to quantum computing, and hence, it is important to develop general methods that do not rely on the details of the systems and can straightforwardly be scaled up on universal quantum computers.

## Supplementary information

Supplementary material

## Data Availability

The data supporting this study (Supplementary Fig. 1) is available at Figshare: 10.6084/m9.figshare.30290674.
