# Supplementary material for: A universal framework for the quantum simulation of Yang–Mills theory
Source: Commun Phys. 2026 Feb 19;9(1):67. doi: 10.1038/s42005-025-02421-6 (PMC12916482; doi:10.1038/s42005-025-02421-6)
Supplement: Supplementary file 1 — Supplementary material [file 42005_2025_2421_MOESM1_ESM.pdf]

Supplementary Information for:

A universal framework for the quantum simulation of Yang–Mills theory

Jad C. Halimeh[a], Masanori Hanada[b], Shunji Matsuura[c], Franco Nori[d], Enrico Rinaldi[e], Andreas Schäfer[f]

[a] Max Planck Institute of Quantum Optics, 85748 Garching, Germany

[a]Department of Physics and Arnold Sommerfeld Center for Theoretical Physics (ASC)
Ludwig Maximilian University of Munich, 80333 Munich, Germany

[a]Munich Center for Quantum Science and Technology (MCQST), 80799 Munich, Germany

[b]School of Mathematical Sciences, Queen Mary University of London
Mile End Road, London, E1 4NS, United Kingdom

[b]qBraid Co., Harper Court 5235, Chicago, IL 60615, United States

[c,e]Interdisciplinary Theoretical & Mathematical Science Program (iTHEMS)
RIKEN, Wako, Saitama 351-0198, Japan

[c]Department of Electrical and Computer Engineering, University of British Columbia
Vancouver, BC V6T 1Z4, Canada

[c]Department of Physics, University of Guelph, ON N1G 1Y2, Canada

[c]Center for Mathematical Science and Advanced Technology, Japan Agency for Marine-Earth Science and Technology, Yokohama 236-0001, Japan

[d,e]Center for Quantum Computing (RQC), RIKEN, Wako, Saitama 351-0198, Japan

[d,e]Theoretical Quantum Physics Laboratory, Cluster of Pioneering Research, RIKEN
Wako, Saitama 351-0198, Japan

[d]Physics Department, University of Michigan, Ann Arbor, MI 48109, United States

[e]Quantinuum K.K., Otemachi Financial City Grand Cube 3F
1-9-2 Otemachi, Chiyoda-ku, Tokyo, Japan

[f]Institute of Theoretical Physics, University of Regensburg
Universitätsstrasse 31, D-93053 Regensburg, Germany

Supplementary Note 1: SU(N) generators and structure constant

In this appendix, we show explicit examples of SU(N) generators τ_α introduced in Matrix Models section of the main text. Our normalization is

$$\mathrm{Tr}(\tau_\alpha \tau_\beta) = \delta_{\alpha\beta}\,. \tag{1}$$

For the SU(2) theory, an explicit example of such generators is obtained by rescaling Pauli matrices as $\frac{\sigma_1}{\sqrt{2}}$, $\frac{\sigma_2}{\sqrt{2}}$, and $\frac{\sigma_3}{\sqrt{2}}$. For the SU(3) theory, we can use the Gell–Mann matrices $\lambda_{1,2,\cdots,8}$ such that

$$\tau_\alpha = \frac{\lambda_\alpha}{\sqrt{2}} \qquad (\alpha = 1, 2, \cdots, 8)\,. \tag{2}$$

The Gell–Mann matrices are defined by

$$\lambda_1 = \begin{pmatrix} 0 & 1 & 0 \\ 1 & 0 & 0 \\ 0 & 0 & 0 \end{pmatrix}, \quad \lambda_2 = \begin{pmatrix} 0 & -\mathrm{i} & 0 \\ \mathrm{i} & 0 & 0 \\ 0 & 0 & 0 \end{pmatrix}, \quad \lambda_3 = \begin{pmatrix} 1 & 0 & 0 \\ 0 & -1 & 0 \\ 0 & 0 & 0 \end{pmatrix},$$
$$\lambda_4 = \begin{pmatrix} 0 & 0 & 1 \\ 0 & 0 & 0 \\ 1 & 0 & 0 \end{pmatrix}, \quad \lambda_5 = \begin{pmatrix} 0 & 0 & -\mathrm{i} \\ 0 & 0 & 0 \\ \mathrm{i} & 0 & 0 \end{pmatrix},$$
$$\lambda_6 = \begin{pmatrix} 0 & 0 & 0 \\ 0 & 0 & 1 \\ 0 & 1 & 0 \end{pmatrix}, \quad \lambda_7 = \begin{pmatrix} 0 & 0 & 0 \\ 0 & 0 & -\mathrm{i} \\ 0 & \mathrm{i} & 0 \end{pmatrix}, \quad \lambda_8 = \frac{1}{\sqrt{3}} \begin{pmatrix} 1 & 0 & 0 \\ 0 & 1 & 0 \\ 0 & 0 & -2 \end{pmatrix}. \tag{3}$$

For SU(N) theory, we can use $\frac{S_{ab}}{\sqrt{2}}$, $\frac{A_{ab}}{\sqrt{2}}$ ($a < b$), and $\frac{D_n}{\sqrt{n(n+1)}}$ ($n = 1, \cdots, N-1$), where

$$(S_{ab})_{ij} \equiv \delta_{ai}\delta_{bj} + \delta_{aj}\delta_{bi}, \quad (A_{ab})_{ij} \equiv \mathrm{i}\,(\delta_{ai}\delta_{bj} - \delta_{aj}\delta_{bi}) \tag{4}$$

and

$$D_n \equiv \mathrm{diag}(1, \cdots, 1, -n, 0, \cdots, 0)\,. \tag{5}$$

The structure constant $f_{\alpha\beta\gamma}$ is defined by

$$[\tau_\alpha, \tau_\beta] = \mathrm{i}\sum_\gamma f_{\alpha\beta\gamma}\tau_\gamma\,. \tag{6}$$

Equivalently,

$$f_{\alpha\beta\gamma} = -\mathrm{i}\cdot \mathrm{Tr}\left([\tau_\alpha, \tau_\beta]\tau_\gamma\right)\,. \tag{7}$$

Combining this expression and trace cyclicity, we can see that $f_{\alpha\beta\gamma}$ is totally antisymmetric.

Supplementary Note 2: Some aspects of gauge symmetry

In this appendix, we discuss a few issues associated with the gauge symmetry, mostly following ref. [1].

When our universal framework is applied to gauge theories—specifically to matrix models and orbifold-lattice constructions—the extended Hilbert space is employed. The use of this extended Hilbert space often causes unnecessary concern due to a widespread misunderstanding that "physical states must be gauge-invariant." We will address this point in Supplementary Note 2, Extended Hilbert space and gauge-invariant Hilbert space. There is no problem as long as the Hamiltonian gauge-invariant. The truncation of the coordinate and momentum bases breaks the gauge symmetry. We argue that the restoration of gauge symmetry can be achieved efficiently in the universal framework.

Extended Hilbert space and gauge-invariant Hilbert space

In the Hamiltonian formulation in the $A_t = 0$ gauge, it is often said that "physical states must be gauge invariant". In fact, however, the gauge-invariant Hilbert space is only one of many equivalent ways to describe physical states [2, 3, 4].

Firstly, let us see how the singlet condition arises. Let us take the canonical partition function as an example, although essentially the same argument holds for real-time evolution. As demonstrated in refs.[5, 6] for the cases of Hermitian and unitary matrix models, the path integral formalism can be rewritten in terms of the operator formalism on the extended Hilbert space as

$$Z(T) = \int [d\phi][dA_t]e^{-S[\phi,A_t]} = \frac{1}{\mathrm{vol}\,\mathcal{G}} \int_{\mathcal{G}} dg \mathrm{Tr}_{\mathcal{H}_{\mathrm{ext}}} \left(\hat{g} e^{-\hat{H}/T} \right) . \tag{8}$$

Here, A_t is the temporal component of the gauge field and ϕ is for all other fields including the spatial component of the gauge field. $\mathcal{G}$ is the local gauge transformation, which is $\mathcal{G} = \prod_{\vec{x}:\mathrm{sites}}[\mathrm{SU}(N)]_{\vec{x}}$ for the case of the lattice gauge theory, $\mathrm{vol}\,\mathcal{G}$ is the volume of $\mathcal{G}$, and $\hat{g}$ acts on the Hilbert space as a gauge transformation corresponding to a group element $g \in \mathcal{G}$. The integral $\int_{\mathcal{G}} dg$ is taken using the Haar measure. $\frac{1}{\mathrm{vol}\,\mathcal{G}} \int_{\mathcal{G}} dg\, \hat{g}$ is a projector from the extended Hilbert space. If one follows the derivation [5, 6] carefully, one can see that g is the Polyakov line and the integration of g is the integration of A_t. The same expression holds for discrete groups, replacing $\mathrm{vol}\,\mathcal{G}$ with the number of elements and $\int dg$ with the sum over all elements. Historically, the first example is the system of N indistinguishable bosons, which can be seen as a gauge theory with S_N gauge symmetry. The projector $\frac{1}{\mathrm{vol}\,\mathcal{G}} \int_{\mathcal{G}} dg\, \hat{g}$ forces us to identify the states related by gauge transformation, like we do in path integral or in classical electrodynamics, or, as Bose and Einstein did, *bosons are not distinguishable*. We can take any state on a gauge orbit [2, 3, 4], and we can also take the linear combination of all the states on the orbit and obtain a single state. The latter leads to another, equivalent expression of the partition function,

$$Z(T) = \mathrm{Tr}_{\mathcal{H}_{\mathrm{inv}}} \left(e^{-\hat{H}/T} \right) . \tag{9}$$

There is no reason that one must use $\mathcal{H}_{\mathrm{inv}}$. By properly avoiding the double counting of euqivalent states, we can describe the same physics by using $\mathcal{H}_{\mathrm{ext}}$.

For quantum simulations, the gauge-invariant Hilbert space poses a serious challenge that must be addressed before it can be employed for large-scale simulations capable of achieving quantum advantage [1]. Namely, due to the non-local nature of the basis, encoding the states and the Hamiltonian is complicated and requires substantial computation on a classical computer,

which becomes a bottleneck because of the exponential growth of the Hilbert-space dimension with the number of qubits. Furthermore, the circuit depth grows exponentially. These issues stand in stark contrast to our universal approach. This makes it difficult to take the lattice size sufficiently large and the lattice spacing sufficiently small.

The key role of gauge symmetry is to introduce a massless vector field compatible with Lorentz symmetry without having negative-norm states [7, 8, 9]. On a lattice, Lorentz symmetry is broken by construction. In the lattice Hamiltonian formulation, Lorentz symmetry is restored when the truncation of the Hilbert space is removed and the continuum limit is taken. The gauge-invariant Hilbert-space approach makes it difficult to take these limits and, as a result, the original motivation for introducing gauge symmetry may be undermined. Somewhat counter-intuitively, by relinquishing exact gauge symmetry at the truncated level, one can better preserve the original motivation for gauge symmetry.

Truncation effect on gauge symmetry

Because our universal framework allows to take the truncation level Λ large, we can remove the truncation effects, including but not limited to those on the gauge symmetry. To demonstrate it, let us study a one-matrix model with the SU(2) gauge group. The coordinate and momebntum operators are $\hat{X} = \frac{1}{\sqrt{2}}\sum_{\alpha=1}^{3}\hat{X}_\alpha\sigma^\alpha$ and $\hat{P} = \frac{1}{\sqrt{2}}\sum_{\alpha=1}^{3}\hat{P}_\alpha\sigma^\alpha$, respectively. Because there are only three bosons, we can use exact diagonalization to study the properties of this model. Unlike multi-matrix models, the gauge-fixed approach is efficient for this case. However, we use the extended Hilbert space here because our motivation is to understand the truncation effect in this approach. For the Hamiltonian, we use

$$\hat{H} = \mathrm{Tr}\left(\frac{1}{2}\hat{P}^2 + \frac{1}{2}\hat{X}^2 + \frac{1}{4}\hat{X}^4\right)\,. \qquad (10)$$

The generators of SU(2) gauge transformation is

$$\hat{G}_\alpha = \sum_{\beta,\gamma} f^{\alpha\beta\gamma}\hat{X}_\beta\hat{P}_\gamma, \qquad (11)$$

or written more explicitly,

$$\hat{G}_1 = \sqrt{2}\left(\hat{X}_2\hat{P}_3 - \hat{X}_3\hat{P}_2\right) ,$$
$$\hat{G}_2 = \sqrt{2}\left(\hat{X}_3\hat{P}_1 - \hat{X}_1\hat{P}_3\right) ,$$
$$\hat{G}_3 = \sqrt{2}\left(\hat{X}_1\hat{P}_2 - \hat{X}_2\hat{P}_1\right) . \tag{12}$$

To estimate the truncation effect, we use the ground-state energy E_0 and the vacuum expectation value of $\sum_\alpha \hat{G}_\alpha^2$. We take the coordinate basis, so that $\hat{X}_\alpha$ take the form of (14) with (16). For $\hat{P}_\alpha$, we use (21), but we use the Fourier transform to express them in the coordinate basis. Therefore, while $\hat{X}_\alpha$ are diagonal, $\hat{P}_\alpha$ are dense matrices.

Supplementary Fig. 1 shows $|E_0(\Lambda) - E_0(\Lambda+2)|$ and $\langle \hat{G}^2 \rangle$ obtained using the ground state, $R = 8$ (fix) and even values of Λ. We can see exponentially fast convergence to the ground state, including an exponential decay of $\langle \hat{G}^2 \rangle$ indicating the exponentially fast restoration of SU(2) symmetry. (Note that the exact gauge symmetry requires $R \to \infty$ as well and hence $\langle \hat{G}^2 \rangle$ does not become exactly zero at finite R.)

Supplementary References

References

[1] Hanada, M., Matsuura, S. M., Mendicelli, E. & Rinaldi, E. Exponential improvement in quantum simulations of bosons (2025).

[2] Hanada, M., Shimada, H. & Wintergerst, N. Color confinement and Bose-Einstein condensation. *JHEP* **08**, 039 (2021). `2001.10459`.

[3] Hanada, M. Bulk geometry in gauge/gravity duality and color degrees of freedom. *Phys. Rev. D* **103**, 106007 (2021). `2102.08982`.

[4] Fliss, J. R., Frenkel, A., Hartnoll, S. A. & Soni, R. M. Minimal Areas from Entangled Matrices (2024). `2408.05274`.

[5] Rinaldi, E. *et al.* Matrix-Model Simulations Using Quantum Computing, Deep Learning, and Lattice Monte Carlo. *PRX Quantum* **3**, 010324 (2022). `2108.02942`.

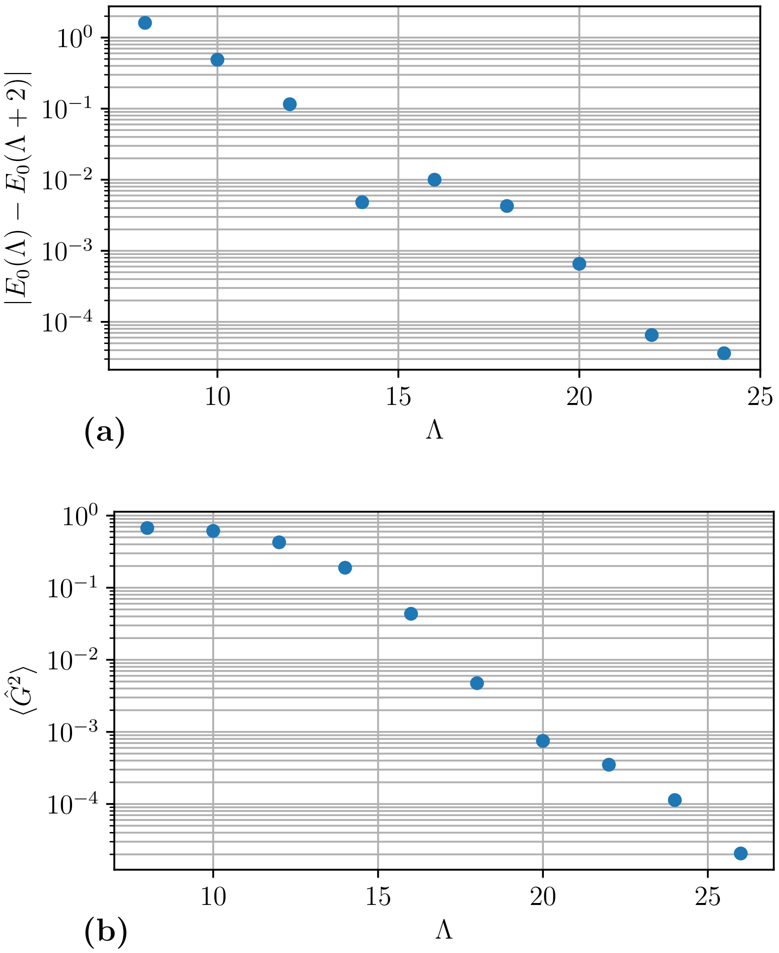

Supplementary Figure 1: Convergence of ground-state observables with truncation level. Panel (a) shows the difference $|E_0(\Lambda) - E_0(\Lambda + 2)|$ at fixed $R = 8$, which is positive for $\Lambda \leq 14$ and negative for $\Lambda \geq 16$. Panel (b) shows the expectation value of $\langle \hat{G}^2 \rangle$ obtained from the ground state at fixed $R = 8$ for various values of Λ.

[6] Gautam, V., Hanada, M., Holden, J. & Rinaldi, E. Linear confinement in the partially-deconfined phase. *JHEP* **03**, 195 (2023). 2208.14402.

[7] Weinberg, S. *The Quantum Theory of Fields*. Vol. 2: Modern Applications (Cambridge University Press, 1995). URL https://books.google.de/books?id=doeDB3_WLvwC.

[8] Peskin, M. E. & Schroeder, D. V. *An Introduction to quantum field theory* (Addison-Wesley, Reading, USA, 1995).

[9] Kugo, T. & Ojima, I. Local Covariant Operator Formalism of Nonabelian Gauge Theories and Quark Confinement Problem. *Prog. Theor. Phys. Suppl.* **66**, 1–130 (1979).
